# Estimating the viscoelastic properties of the human brain at 7 T MRI using intrinsic MRE and nonlinear inversion

**DOI:** 10.1002/hbm.26524

**Published:** 2023-11-01

**Authors:** Marius Burman Ingeberg, Elijah Van Houten, Jaco J. M. Zwanenburg

**Affiliations:** ^1^ Center for Image Sciences University Medical Center Utrecht Utrecht The Netherlands; ^2^ University de Sherbrooke Sherbrooke Quebec Canada

**Keywords:** DENSE, elastography, high field, iMRE, intrinsic, shear stiffness, viscoelastic properties

## Abstract

Intrinsic actuation magnetic resonance elastography (MRE) is a phase‐contrast MRI technique that allows for in vivo quantification of mechanical properties of the brain by exploiting brain motion that arise naturally due to the cardiac pulse. The mechanical properties of the brain reflect its tissue microstructure, making it a potentially valuable parameter in studying brain disease. The main purpose of this study was to assess the feasibility of reconstructing the viscoelastic properties of the brain using high‐quality 7 T MRI displacement measurements, obtained using displacement encoding with stimulated echoes (DENSE) and intrinsic actuation. The repeatability and sensitivity of the method for detecting normal regional variation in brain tissue properties was assessed as secondary goal. The displacement measurements used in this analysis were previously acquired for a separate study, where eight healthy subjects (27 ± 7 years) were imaged with repeated scans (spatial resolution approx. 2 mm isotropic, temporal resolution 75 ms, motion sensitivity 0.35 mm/2*π* for displacements in anterior–posterior and left–right directions, and 0.7 mm/2*π* for feet–head displacements). The viscoelastic properties of the brain were estimated using a subzone based non‐linear inversion scheme. The results show comparable consistency to that of extrinsic MRE between the viscoelastic property maps obtained from repeated displacement measurements. The shear stiffness maps showed fairly consistent spatial patterns. The whole‐brain repeatability coefficient (RC) for shear stiffness was (mean ± standard deviation) 8 ± 8% relative to the mean whole‐brain stiffness, and the damping ratio RC was 28 ± 17% relative to the whole‐brain damping ratio. The shear stiffness maps showed similar statistically significant regional trends as demonstrated in a publicly available atlas of viscoelastic properties obtained with extrinsic actuation MRE at 50 Hz. The damping ratio maps showed less consistency, likely due to data‐model mismatch of describing the brain as a viscoelastic material under low frequencies. While artifacts induced by fluid flow within the brain remain a limitation of the technique in its current state, intrinsic actuation based MRE allow for consistent and repeatable estimation of the mechanical properties of the brain. The method provides enough sensitivity to investigate regional variation in such properties in the normal brain, which is likely sufficient to also investigate pathological changes.

## Graphical abstract

The mechanical properties of brain reflect its tissue microstructure, making it a potentially valuable parameter in studying brain disease. Intrinsic actuation based MRE allow for consistent and repeatable estimation of the mechanical properties of the brain and provides enough sensitivity to investigate regional variation in such properties.

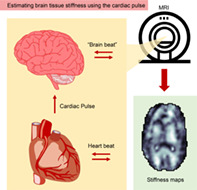

## 1 INTRODUCTION

Magnetic resonance elastography (MRE) (Muthupillai et al., 1995) is a phase‐contrast MRI technique that allows for in vivo quantification of mechanical properties within any tissue. It shows potential as an additional contrast mechanism in the assessment and diagnosis of pathologies that affect tissue microstructure (Glaser et al., 2012; Hiscox et al., 2016; Rouvière et al., 2006; Yin et al., 2007). Recently, there has been increasing interest in applying MRE to the brain, for two principal reasons. First, MRE may provide an additional perspective into brain function, given recent studies demonstrating the link between tissue mechanics and neuronal activity (Barnes et al., 2017; Forouhandehpour et al., 2021; Goriely et al., 2015; Lan et al., 2020; Schwarb et al., 2016). Secondly, it provides a non‐invasive approach to constructing detailed maps of the mechanical properties of the brain, which has previously been exceedingly difficult. Additionally, it allows for the quantification of mechanical properties while the brain is within its natural environment. In contrast, ex vivo attempts at quantifying such properties faces many challenges due to the inconsistent nature of extracted soft tissue, leading to contradictory results (Budday et al., 2020).

Typically, MRE uses a mechanical actuator to produce mechanical waves within the tissue of interest in order to produce displacements that can be imaged using phase‐contrast techniques. The resulting displacement fields can then be used to reconstruct the mechanical properties of the tissue via various types of *inversion*, or image reconstruction. As previously shown, it is possible to reconstruct the mechanical properties of the brain without the use of an extrinsic driver (Herthum et al., 2021; Hirsch et al., 2013; Weaver et al., 2012; Zorgani et al., 2015). This approach is referred to as “intrinsic actuation” and uses the brain motions that arise naturally due to the cardiac pulse, which can be measured given a sufficiently sensitive MRI acquisition. Intrinsic actuation MRE has a few important benefits. First, it eliminates the necessity of external actuators, significantly simplifying the method of acquisition, which in turn increases the availability of the method. Second, it circumvents the problem of poor shear wave penetration through the skull (Weaver et al., 2012). Finally, the use of the cardiac pulse signal for MRE actuation leads to a mechanical profile of the brain in its *natural* state, rather than in an artificial vibration state.

Since the first attempts at intrinsic actuation MRE, there has been significant improvement to the acquisition of cardiac‐induced brain deformation. Initial intrinsic acquisition methods employed traditional phase‐contrast velocity measurements, which are not very sensitive to the low velocities of brain tissue motion during the cardiac cycle, which are typically below 1 mm/s. Over time, MRI sequences dedicated to quantifying cardiac‐induced brain tissue motion emerged, enabling more sensitive measurements. Specifically, the acquisition sequence displacement encoding with stimulated echoes (DENSE), originally developed for measuring motion in the myocardium (Aletras et al., 1999), can be adapted to measure subtle brain tissue motion (Reese et al., 2002; Soellinger et al., 2009). This sequence has recently been further optimized and adapted to exploit the benefits of 7 T MRI. (Adams et al., 2019, 2020; Sloots et al., 2020, 2021). Such data, however, has not yet been tested in reconstructing the mechanical properties of the brain. The main purpose of this work is thus to assess the feasibility of reconstructing the mechanical properties of the brain using 7 T displacement measurements, obtained using DENSE acquisition and intrinsic actuation. As secondary goals, we want to verify that the repeatability of the DENSE measurements is maintained after inversion as well as exploring the sensitivity of the method for detecting normal variation in brain tissue properties. This is done by conducting a regional analysis where we compare regional mechanical properties to those provided in a publicly available atlas (in standard‐space) of viscoelastic properties obtained with extrinsic actuation (which we denote as the *extrinsic viscoelastic property atlas in standard space*, or *EVPASS*) (Hiscox et al., 2020).

## 2 METHOD

### 2.1 Utilized brain motion data

The displacement measurements used in this analysis were previously acquired by Adams et al. (2020). Details of their method of acquisition and data processing can be found in the original paper (Adams et al., 2020). We will briefly summarize the method and resulting data in the following section. Subjects gave written‐informed consent for participation in this study, which was approved by the ethical review board of our institution.

Eight healthy young subjects (three females, mean age: 27 ± 6 years) were imaged using the DENSE sequence in a 7 T MR scanner (Philips Healthcare), where a 3D EPI readout scheme was used. Repeated scans were performed to assess the repeatability of the measurements, with the subjects exiting the scanner for a maximum of 10 min between scans. A pulse oximeter was used to sync the DENSE measurements to the cardiac cycle by means of retrospective gating. The acquired resolution was 1.95 mm × 1.95 mm × 2.2 mm, with 20 phases (time points) reconstructed per cardiac cycle. Three acquisitions were performed with displacement encoding (*D*_ENC_) sensitivity of 0.35, 0.175, and 0.175 mm in the FH, AP, and RL directions, respectively (i.e., displacement *D*_ENC_ causes a phase shift of *π*). The acquisition time for a heart rate of 60 beats/min was 2:24 min per motion encoding direction. T1‐weighted images were also obtained during both scans, with an acquired resolution of 0.93 mm × 0.93 mm × 1.0 mm. The Computational Anatomy Toolbox (Jena University Hospital, Departments of Psychiatry and Neurology) extension for SPM12 (Wellcome Trust Centre for Neuroimaging, University College London) was used to create a brain tissue mask that excludes large blood vessels. The toolbox was additionally used to generate gray matter (GM) and white matter (WM) probability maps, as well as cerebrospinal fluid (CSF) probability maps from T1‐weighted images. Larger intracranial blood vessels induced high intensities in the T1‐weighted images, affecting the segmentation. They were consequently removed from the GM and WM probability maps using morphological area opening. Additionally, physiological motion that is not synchronized with the triggering device results in inter‐shot phase variability and consequently artifacts. Such artifacts were detected through the use of SNR maps, which were obtained by dividing the magnitude image at the end of the cardiac cycle (which contained the majority of artifacts) by the temporal standard deviation. The SNR maps could then be thresholded to create artifact‐free masks, which were combined with the GM and WM masks using logical conjunction. A more detailed description of the above‐mentioned methodology can be found in the original article (Adams et al., 2020).

AP and FH magnitude images were rigidly registered to the RL magnitude images (Klein et al., 2010) to account for subject motion between the three acquisitions, as described before (Adams et al., 2020). The last RL image of the cardiac cycle was then registered to the T1‐weighted image for each subject, using non‐linear registration. All images were then transformed to the T1‐weighted image orientation and resolution using the non‐linear registration parameters obtained from the RL to T1 registration and linear interpolation. All image registrations were performed using Elastix (Klein et al., 2010).

### 2.2 Mechanical property estimation

The mechanical properties were estimated using a subzone based non‐linear inversion (NLI) scheme (McGarry et al., 2012; Van Houten et al., 1999, 2001). In order to perform such inversions, quadratic hexahedral finite element models (FEM) of the subject's brains are generated from the respective displacement measurements, where an isotropic mesh size of 2.0 mm was used. The reconstruction is formulated as a constrained optimization task where the objective is to minimize the difference between the measured tissue displacements and displacements obtained using the computational finite element model.

A viscoelastic model of the brain is used, which recovers the complex shear modulus, $G^{*}=G^{\prime }+{\mathrm{iG}}^{\prime \prime }$, where $G^{\prime }$ is the shear storage modulus and $G^{\prime \prime }$ is the shear loss modulus. Additionally, the lambda‐modulus, $\text{λ}=\frac{2\nu G^{*}}{1-2\nu }$, where ν is the Poisson ratio, is recovered with a 20 cm isotropic mesh resolution, such that one single value for λ is recovered for the entire brain volume. The equation of motion for a heterogeneous viscoelastic material expressed in the frequency domain is given by

(1) $$\nabla ∙G^{*}\left(\nabla u+\nabla u^{T}\right)+\nabla \left(\lambda \nabla ∙u\right)=-\rho \omega^{2}u+F,$$

with u being the 3D complex‐valued motion amplitude, ρ the density, ω the actuation frequency, and F the body forces (which are assumed to be 0 such that F=0). As discussed more thoroughly by McGarry et al. (2019), the elastic forces on the left‐hand side of Equation (1) are much larger than the inertial forces described by the right‐hand side. Equation (1) therefore reduces to

(2) $$\nabla ∙G^{*}\left(\nabla u+\nabla u^{T}\right)+\nabla \left(\lambda \nabla ∙u\right)\sim 0.$$

All terms on the left‐hand side of Equation (2) contain $G^{*}$, which means that a constant multiplier $g_{0}$ can be factored out as $G^{*}$=$g_{0}G_{0}^{*}$ and $\lambda =g_{0}\lambda_{0}=\frac{2\nu G_{0}^{*}}{1-2\nu }$, such that

(3) $$g_{0}\left[\nabla ∙G_{0}^{*}\left(\nabla u+\nabla u^{T}\right)+\nabla \left(\lambda_{0}\nabla ∙u\right)\right]\sim 0.$$

Because $g_{0}$ is arbitrarily scalable and has no effect on the solution, it is only possible to recover relative distributions of $G^{*}$ for the low frequencies exhibited in intrinsic actuation. Due to this effect, a lower bound of 100 Pa is applied to $G^{*}$ and λ to ensure non‐negative solutions.

The FEM property distributions are updated iteratively such that the model displacements approach the measured displacements. A subzone size of 20 mm^3^ with 15% overlap between zones was used, and 800 global iterations were performed for each subject in order to assure convergence. The hexahedral finite element meshes contained (mean ± std) 157,730 ± 15,420 nodes. Finally, the shear stiffness $\tilde{\mu }=2{\left|G^{*}\right|}^{2}/\left(G^{\prime }+\left|G^{*}\right|\;\right)$ and the damping ratio $\xi =G^{\prime \prime }/2G^{\prime }$ are computed and reported to stay consistent with literature. The shear stiffness $\tilde{\mu }$ has been denoted with a tilde to reflect the non‐unique, relative nature of these intrinsic actuation viscoelastic solutions.

### 2.3 Fluid flow artifact removal

The DENSE protocol is tuned to measure small tissue deformations. Consequently, large motions from fluid flow within the brain, such as larger blood vessels and CSF, can give incorrect values when measured, potentially inducing artifacts. Such artifacts can appear in mechanical property distributions as large *hotspots*, perturbing the results in the vicinity of the artifact. The majority of such hotspots appear in the periphery of the brain around the subarachnoid space which contains large quantities of CSF. As the cardiac‐induced brain pulsations have the lowest amplitudes in such areas, the areas with large motions due to CSF can be determined with more certainty.

A heuristic method to deal with such hotspots in the periphery was implemented by masking voxels in the DENSE measurements that contained unreasonably high displacements. The masking of fluid‐flow dominated voxels was optimized by simultaneously minimizing masking of brain tissue in two ways. First, the masking was spatially limited to the peripheral area of the brain as it contained the vast majority of fluid flow related hotspots. Secondly, a suitable threshold for (a) the spatial extent of what is defined as the periphery and (b) for the threshold motion amplitude for masking, was empirically determined by performing a number of trial runs with variable settings for multiple subjects. The settings which appeared to have the most reasonable trade‐off between removal of erroneous fluid flow related displacement measurements and unwanted removal of voxels representing true tissue displacement measurements was found to be: a periphery defined by a 5 voxel deep layer (i.e., 5 mm) around the exterior boundary of the brain and a masking threshold of eight times the standard deviation of the displacement distribution within the brain. Any *stranded* voxels that ended up disconnected from the bulk of the brain volume were removed, and an additional one layer of voxel erosion was performed on the resulting mask.

### 2.4 Regional analysis

Regional differences within the brain were evaluated to explore the capability of the method to assess normal regional variation in tissue mechanical parameters. We investigated the variation in shear stiffness and damping ratio in the same regions of interest as is done in the EVPASS for reference. These regions are divided over cortical GM, subcortical GM, and white matter tracts (WMT), and were obtained from various standard‐space MNI atlases, specified below.

#### 2.4.1 Gray matter

The MNI‐ICBM2009c nonlinear symmetric 1 mm atlas (Manera et al., 2020) was used to identify cortical and subcortical GM. The cortical GM ROIs include the following 12 regions: cuneus (CN), fusiform gyrus (FSG), inferior temporal cortex (ITC), lateral occipital cortex (LaO), lingual occipital cortex (LiO), precuneus (PCN), postcentral cortex (POST), rostral middle frontal cortex (RMF), precentral cortex (PRE), superior frontal cortex (SFC), superior parietal cortex (SPC), and superior temporal cortex (STC). Similarly, the six subcortical GM ROIs include the amygdala (AM), caudate (CA), hippocampus (HC), pallidum (PA), putamen (PU), and thalamus (TH). Each regional mask was individually eroded by 1 voxel to obtain a more conservative estimate of the locations of the regions, thereby avoiding partial volume effects that occur at the boundaries.

#### 2.4.2 White matter

Twelve WMT ROIs were identified, eight of which were obtained from the JHU‐ICBM‐tracts 2 mm atlas, thresholded at 25%. These include the anterior thalamic radiation (ATR), corticospinal tract (CST), major forceps (FMa), minor forceps (FMi), inferior frontal occipital fasciculus (IFOF), inferior longitudinal fasciculus (ILF), superior longitudinal fasciculus (SLF), and uncinate fasciculus (UN). The other four regions were obtained from the JHU‐ICBM‐labels 2 mm atlas, including corpus callosum (CC), corona radiata (CRa), fornix (FX), and posterior thalamic radiation (PTR). Each regional mask was individually eroded by 1 voxel.

#### 2.4.3 Global regions of interest

Each global region of interest was also investigated separately, where the cortical GM and subcortical GM masks were created by combining all investigated cortical and subcortical GM regions respectively. GM probability distributions were additionally used in combination with the regional masks, with a threshold of 90%. WM masks were generated by thresholding the white matter probability masks at 90%.

#### 2.4.4 Region extraction

To extract the regions from the shear stiffness and damping ratio maps, the T1‐weighted images (which were in the same space as the DENSE magnitude images) of each subject were non‐linearly registered to the MNI‐ICBM2009c non‐linear average template, using Elastix (Klein et al., 2010). Two levels of resolution were used, with 250 iterations per resolution. Prior to the non‐linear registration, each T1‐weighted image was aligned to the MNI template by first performing a rigid registration for 100 iterations for 4 levels of resolution, followed by an affine registration for 200 iterations for one level of resolution. The resulting transformation matrices were then applied to the shear stiffness and the damping ratio distributions as well as grey and white matter probability distributions using nearest neighbor interpolation. Additionally, the JHU‐ICBM atlases were interpolated to the resolution of the MNI‐ICBM2009c non‐linear average template using nearest neighbor interpolation such that the parameter distributions and all atlases have the same resolution. Each atlas was then used to identify each ROI in the shear stiffness and damping ratio distribution for each subject. Repeated scans are averaged such that there is only one data point per subject.

#### 2.4.5 Statistical analysis

Post hoc pairwise comparisons were performed to examine regional differences in the shear stiffness and damping ratio by using General Linear mixed models. Bonferroni corrections were used to counteract the multiple comparisons problem. A value of p<0.05 was used as the threshold for statistically significant effects. All statistical analyses were performed using the glmm package for R.

### 2.5 Atlas comparison

To assess differences between the extrinsic and intrinsic actuation approaches more thoroughly, the shear stiffness and damping ratio templates presented in the EVPASS, obtained by averaging spatially normalized parameter maps from 134 participants, were compared voxel‐by‐voxel with the equivalent mean distributions presented in this analysis. To do so, the EVPASS distributions were interpolated using nearest neighbor interpolation to match the resolution of the MNI‐ICBM2009c template. To account for the arbitrary scaling factor of the intrinsic results, all distributions were normalized by their global mean value throughout the brain volume, and maps representing the voxel‐wise differences were obtained by computing the ratio between the respective distributions. Finally, the difference in regional variation of the shear stiffness and damping ratio between the two approaches was explored by directly comparing the regional means obtained in the intrinsic analysis presented here with the ones presented in the EVPASS. This was done by plotting the regional means obtained with both methods together. Due to the difference in scaling between the two methods, the y‐axes are scaled using global mean values throughout the brain volume such that the relative values can be evaluated.

### 2.6 Repeatability analysis

To assess the repeatability of the measurements, a voxel‐wise Bland–Altman analysis (Bland & Altman, 1986) was performed on each subject for the shear stiffness and damping ratio. The analysis was performed in MNI space to assess the repeatability in the cortical GM, the subcortical GM, and the WM separately using the global ROI masks. A subject‐wise Bland–Altman analysis was additionally performed for the mean shear stiffness and damping ratio in each global ROI. Finally, the repeatability coefficient (RC) and coefficient of variation (CV) were calculated for each subject and global ROI.

## 3 RESULTS

### 3.1 Mechanical property distributions

**FIGURE 1:**
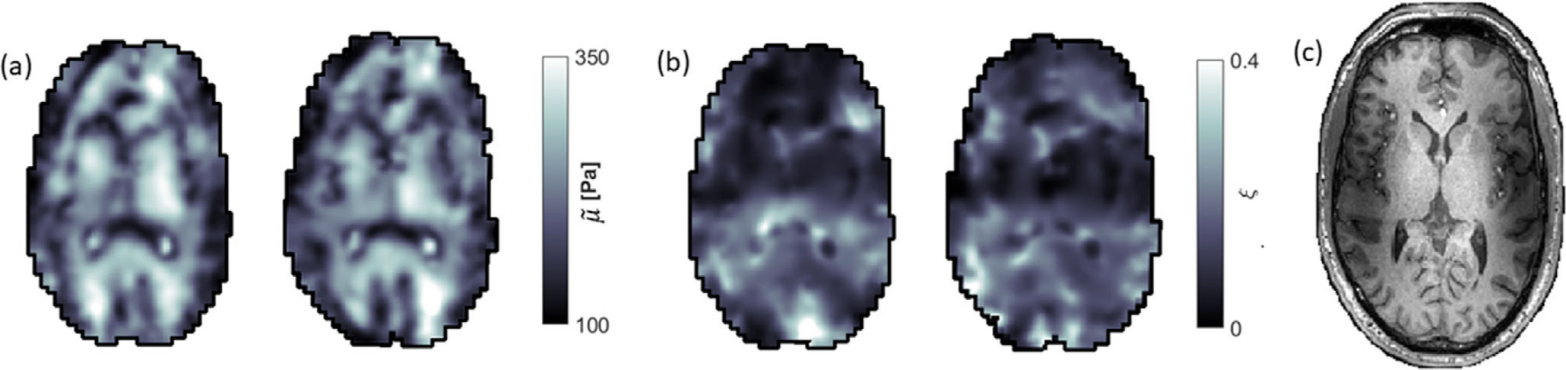
Representative axial slices of the (a) shear stiffness, $\tilde{\mu }$, and the (b) damping ratio, ξ, for one subject along with repeat scans that have been co‐registered to the initial scan. A T1‐weighted image of the equivalent slice is shown in (c). Consistent spatial patterns can be observed throughout the property maps, and the repeated scans show strong structural similarity to that of the original scans, especially for the shear stiffness. The damping ratio maps show some consistency between repeated scans but only limited similarity to the anatomical scan, likely due to data‐model mismatch and the noise propagation of the two reconstructed parameters, *G*′ and *G*″ into this composite parameter (see Section 4). The dark regions observed in the shear stiffness maps largely correspond to CSF‐tissue boundaries. Large‐valued hotspots can be seen in the repeated scan of the shear stiffness map, and in the original scan of the damping ratio map. The shear stiffness has been denoted with a tilde to the relative nature of the recovered solutions (see Section 2).

The viscoelastic property maps were successfully reconstructed in all subjects, including the repeated scans. The total change in property values between final iterations of the non‐linear inversion was less than 0.053% for all subjects. An average of 3.45% (2.35%–6.13%) of the total amount of voxels per subject were masked to suppress fluid flow artifacts. Figure 1 shows a representative axial slice in one subject of the shear stiffness and the damping ratio, along with the equivalent slice in the repeated scan which has been co‐registered to the original scan. The equivalent axial slices for all subjects are presented in Figure S1 (shear stiffness) and Figure S2 (damping ratio). Consistent spatial patterns can be observed throughout the property maps, and general structural features of the brain can be identified. The shear stiffness maps of the repeated scans show strong structural similarity to that of the original scans, while the damping ratio distribution generally exhibits more variability. Though the property maps are generally symmetric (average difference between symmetric brain regions was 0.2% for the shear stiffness and 6.5% for the damping ratio), they remain affected by the existence of hotspots, as no fluid flow masking was applied to the interior of the brain volume. The mean lambda modulus (single value per brain) over all subjects was 100.34 ± 0.27 Pa for the initial scans, and 100.22 ± 0.12 Pa for the repeated scans.

### 3.2 Regional analysis

**FIGURE 2:**
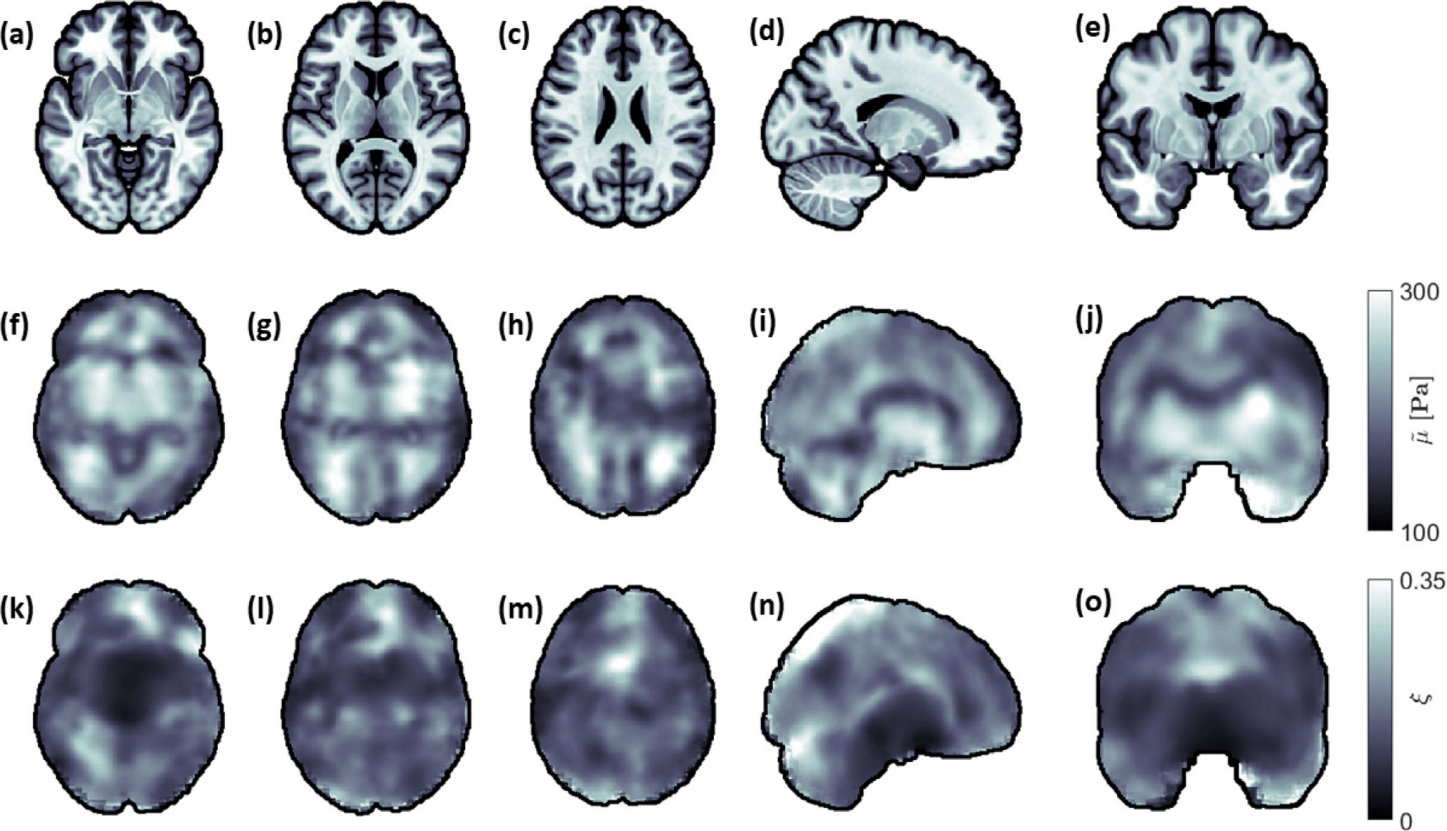
Transverse (a–c), sagittal (d), and coronal (e) anatomical representations of the brain in common space (MNI‐ICBM2009c template), and respective shear stiffness (f–j) and damping ratio (k–o) maps, obtained from averaging over all subjects and repeats. Each individual parameter map has been registered to the MNI‐ICBM2009c brain atlas. The shear stiffness distributions display clear spatial patterns and large regional variability, where many features of the brain can be identified. The damping ratio distributions exhibit less spatial definition. Discretization artifacts can be observed along the boundary of the brain due to the masking of voxels containing fluid flow. The shear stiffness has been denoted with a tilde to the relative nature of the recovered solutions.

The mean over all subjects and repeats for the $\tilde{\mu }$ and ξ distributions are shown in Figure 2, where each image has been registered to the MNI‐ICBM2009c non‐linear template. The MNI‐ICBM2009c template is also included in Figure 2 for visual comparison. The shear stiffness distribution shows high symmetry, clear spatial patterns, and large regional variability in all presented slices. Many structures of the brain can be identified that largely match anatomical structures seen in the MNI template. Some hotspots can be observed, such as in the lower left and top right regions in Figure 2k. The damping ratio distributions generally show less symmetry and spatial definition. Due to the masking of voxels containing fluid flows in the motion data, the boundaries of the finite element models used in NLI reconstruction can differ between subjects. For this reason, discretization artifacts can be observed along the periphery of the brain, particularly in the coronal slices (Figure 2j,o).

All ROIs were successfully identified within the parameter maps in each subject. The size of each region per MRE measurement is presented in Tables S1–S3; reported in number of voxels of 2 mm isotropic resolution (approximately original resolution of DENSE measurements). The variation in size between equivalent ROIs is due to the different levels of artifact removal performed throughout the analysis, where certain voxels are masked.

#### 3.2.1 Global regions of interest

**FIGURE 3:**
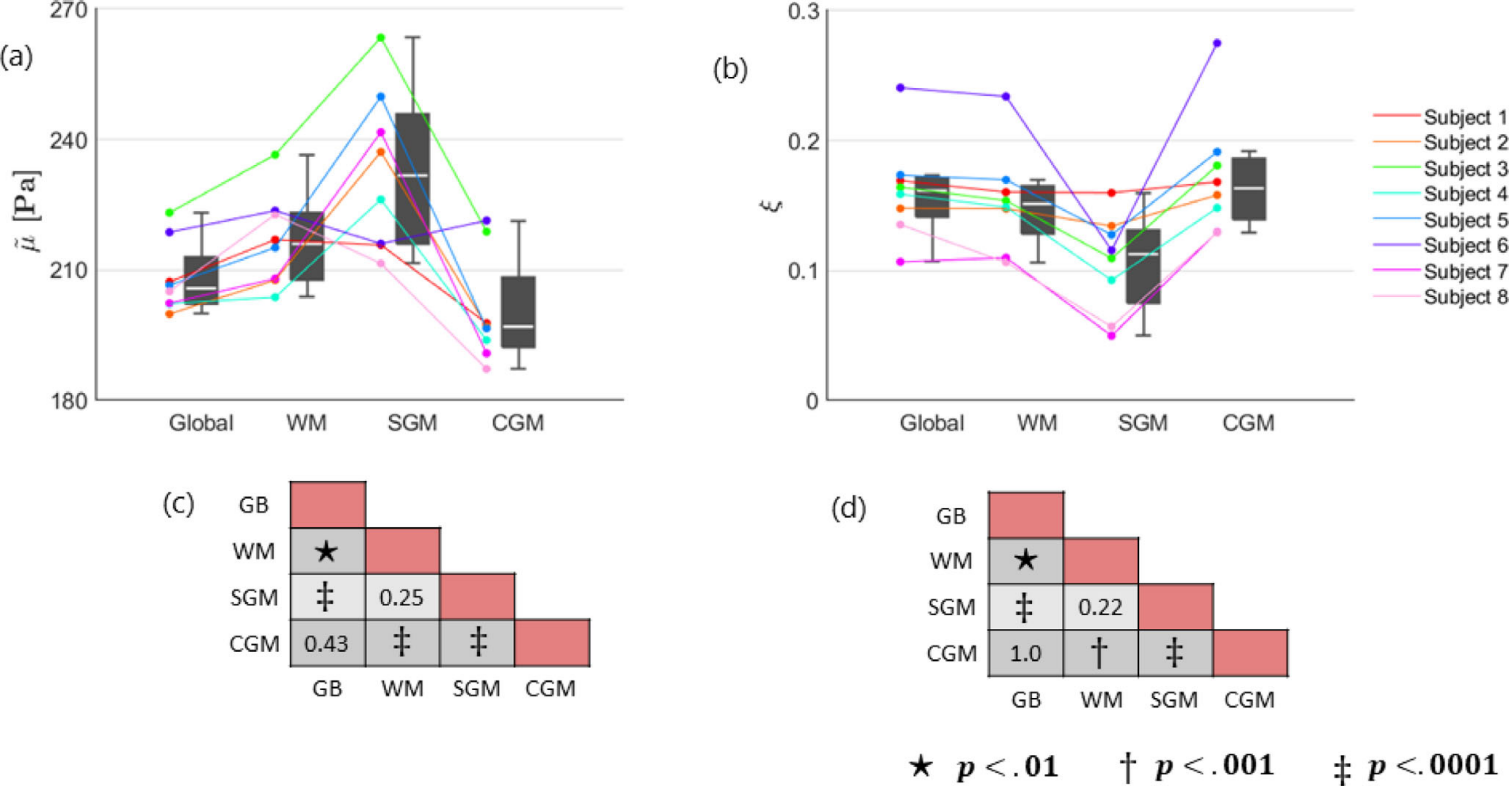
Boxplots alongside individual trend lines for each subject and significance charts in selected global ROIs for (a) shear stiffness, $\tilde{\mu }$, and (b) damping ratio, ξ. Each dot represents the mean within one region for one subject. The white line within the boxplots correspond to the median and the upper and lower end of the boxplots represent the 25th and 75th quartiles. The whiskers illustrate the maximum and minimum values that are not outliers, where outliers are values which lie at least 1.5∙IQR away from the upper or lower quartiles. The significance charts for the (c) shear stiffness and (d) damping ratio were determined by utilizing a general linear mixed model and performing a post‐hoc analysis which was adapted for multiple comparisons using Bonferroni correction. Regions that are significantly different are indicated with bold text, and the symbols: ⋆, †, or ‡, depending on their level of significance. The shear stiffness has been denoted with a tilde to indicate the relative nature of the recovered solutions.

The mean and standard deviation over all subjects within each global ROI are provided in Table 1. The shear stiffness showed regional differences that were consistent between subjects, as visualized in plot (a) of Figure 3. Likewise, the damping ratio is presented in plot (b) of Figure 3. The pairwise comparisons of each ROI are presented as significance charts for (c) the shear stiffness and (d) the damping ratio.

**TABLE 1:** Mean and standard deviation of stiffness parameters in WMT ROIs.

	Shear stiffness, $\tilde{\mu }$ (Pa)	Damping ratio, ξ
Global	208 ± 14	0.162 ± 0.042
WM	217 ± 15	0.154 ± 0.043
Subcortical GM	213 ± 21	0.106 ± 0.039
Cortical GM	200 ± 14	0.173 ± 0.053

#### 3.2.2 White matter tracts

**FIGURE 4:**
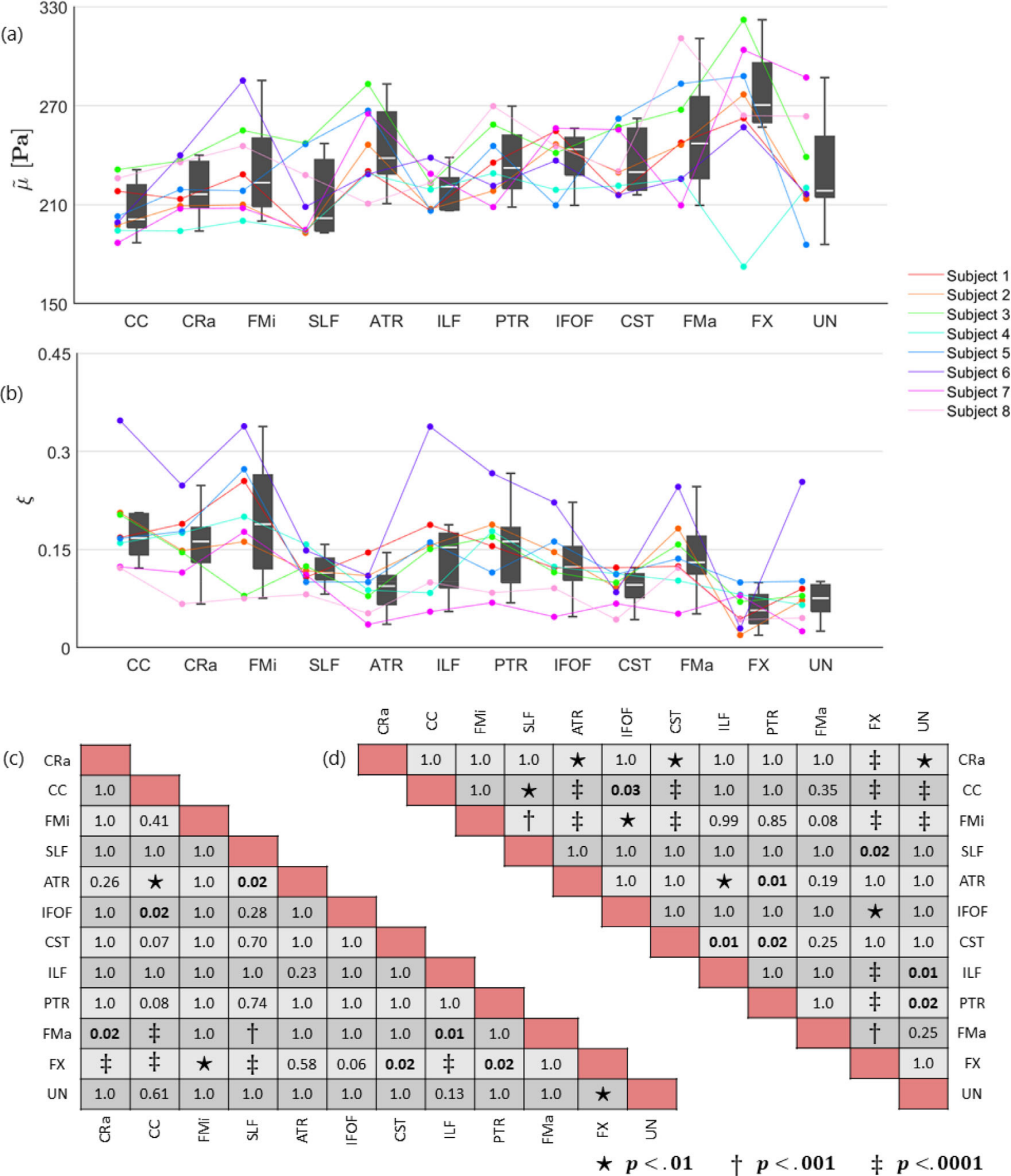
Boxplots alongside individual trend lines for each subject and significance charts in selected WMT ROIs for (a) shear stiffness, $\tilde{\mu }$, and (b) damping ratio, ξ. Each dot represents the mean within one region for one subject. The regions are sorted by number of voxels such that the largest regions appear on the left. The white line within the boxplots correspond to the median and the upper and lower end of the boxplots represent the 25th and 75th quartiles. The whiskers illustrate the maximum and minimum values that are not outliers, where outliers are values which lie at least 1.5∙IQR away from the upper or lower quartiles. The significance charts for the (c) shear stiffness and (d) damping ratio were determined by utilizing a general linear mixed model and performing a post‐hoc analysis which was adapted for multiple comparisons using Bonferroni correction. Regions that are significantly different are indicated with bold text, and the symbols: ⋆, †, or ‡, depending on their level of significance. The shear stiffness has been denoted with a tilde to indicate the relative nature of the recovered solutions. The abbreviations for the regions can be found in the legend of Table 2.

The mean and standard deviation over all subjects within each WMT region are provided in Table 2. The shear stiffness showed regional differences that were consistent between subjects, as visualized in plot (a) of Figure 4. Likewise, the damping ratio is presented in plot (b) of Figure 4 but exhibits overall less consistent regional differences between subjects. The pairwise comparisons of each ROI is presented as significance charts for (c) the shear stiffness and (d) the damping ratio.

**TABLE 2:** Mean and standard deviation of stiffness parameters in WMT ROIs.

	Shear stiffness, $\tilde{\mu }$ (Pa)	Damping ratio, ξ
CC	207 ± 16	0.187 ± 0.072
CRa	219 ± 16	0.158 ± 0.054
FMi	231 ± 29	0.196 ± 0.092
SLF	213 ± 24	0.119 ± 0.025
ATR	245 ± 25	0.090 ± 0.035
IFOF	219 ± 12	0.154 ± 0.087
CST	236 ± 21	0.153 ± 0.064
ILF	239 ± 17	0.123 ± 0.051
PTR	236 ± 19	0.092 ± 0.026
FMa	252 ± 34	0.140 ± 0.057
FX	268 ± 45	0.058 ± 0.028
UN	230 ± 32	0.92 ± 0.070

Abbreviations: ATR, anterior thalamic radiation; CC, corpus callosum; CRa, corona radiata; CST, corticospinal tract; FMa, major forceps; FMi, minor forceps; FX, fornix; IFOF, inferior frontal‐occipital fasciculus; ILF, inferior longitudinal fasciculus; PTR, posterior thalamic radiation; SLF, superior longitudinal fasciculus; UN, uncinate.

#### 3.2.3 Cortical gray matter

**FIGURE 5:**
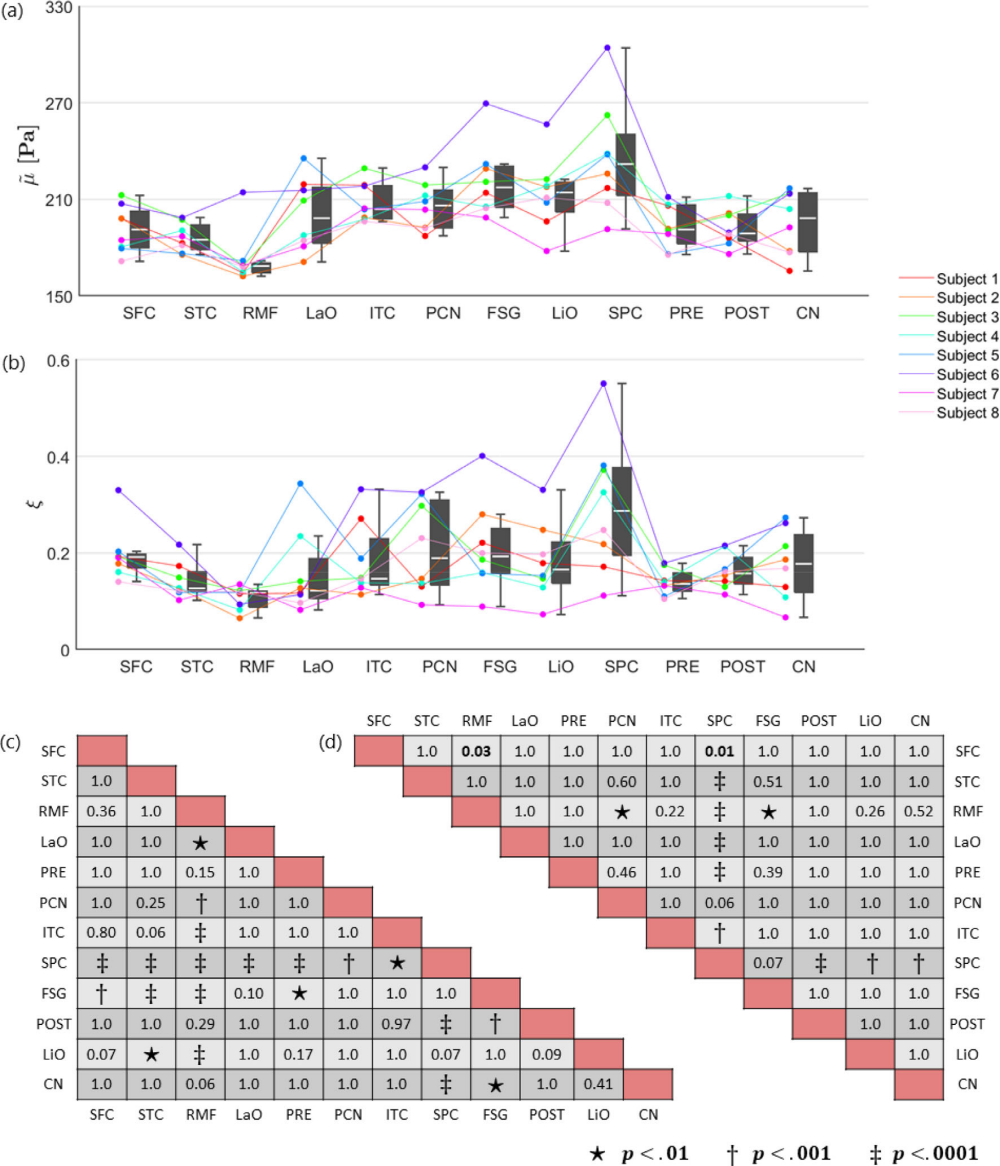
Boxplots alongside individual trend lines for each subject and significance charts in selected cortical GM ROIs for (a) shear stiffness, $\tilde{\mu }$, and (b) damping ratio, ξ. Each dot represents the mean within one region for one subject. The regions are sorted by number of voxels such that the largest regions appear on the left. The white line within the boxplots correspond to the median and the upper and lower end of the boxplots represent the 25th and 75th quartiles. The whiskers illustrate the maximum and minimum values that are not outliers, where outliers are values which lie at least 1.5∙IQR away from the upper or lower quartiles. The significance charts for the (c) shear stiffness and (d) damping ratio were determined by utilizing a general linear mixed model and performing a post‐hoc analysis which was adapted for multiple comparisons using Bonferroni correction. Regions that are significantly different are indicated with bold text, and the symbols: ⋆, †, or ‡, depending on their level of significance. The shear stiffness has been denoted with a tilde to indicate the relative nature of the recovered solutions. The abbreviations for the regions can be found in the legend of Table 3.

The mean and standard deviation over all subjects within each cortical GM region are provided in Table 3. The shear stiffness showed regional differences that were consistent between subjects, as visualized in plot (a) of Figure 5. Likewise, the damping ratio is presented in plot (b) of Figure 5 but exhibits overall less consistent regional differences between subjects. The pairwise comparison of each ROI is presented as significance charts for (c) the shear stiffness and (d) the damping ratio.

**TABLE 3:** Mean and standard deviation of stiffness parameters in cortical GM ROIs.

	Shear stiffness, $\tilde{\mu }$ (Pa)	Damping ratio, ξ
SFC	191 ± 15	0.198 ± 0.057
STC	186 ± 9	0.142 ± 0.037
RMF	173 ± 17	0.106 ± 0.024
LaO	200 ± 23	0.157 ± 0.089
ITC	208 ± 12	0.183 ± 0.078
PCN	206 ± 15	0.210 ± 0.094
FSG	222 ± 23	0.212 ± 0.094
LiO	214 ± 23	0.182 ± 0.079
SPC	236 ± 35	0.297 ± 0.140
PRE	193 ± 14	0.140 ± 0.027
POST	192 ± 12	0.162 ± 0.037
CN	195 ± 20	0.176 ± 0.073

Abbreviations: CN, cuneus; FSM, fusiform gyrus; ITC, inferior temporal cortex; LaO, lateral occipital cortex; LiO, lingual occipital cortex; PCN, precuneus; POST, postcentral cortex; RMF, rostral middle frontal cortex; SFC, superior frontal cortex; SPC, superior parietal cortex; STC, superior temporal cortex.

#### 3.2.4 Subcortical gray matter

**FIGURE 6:**
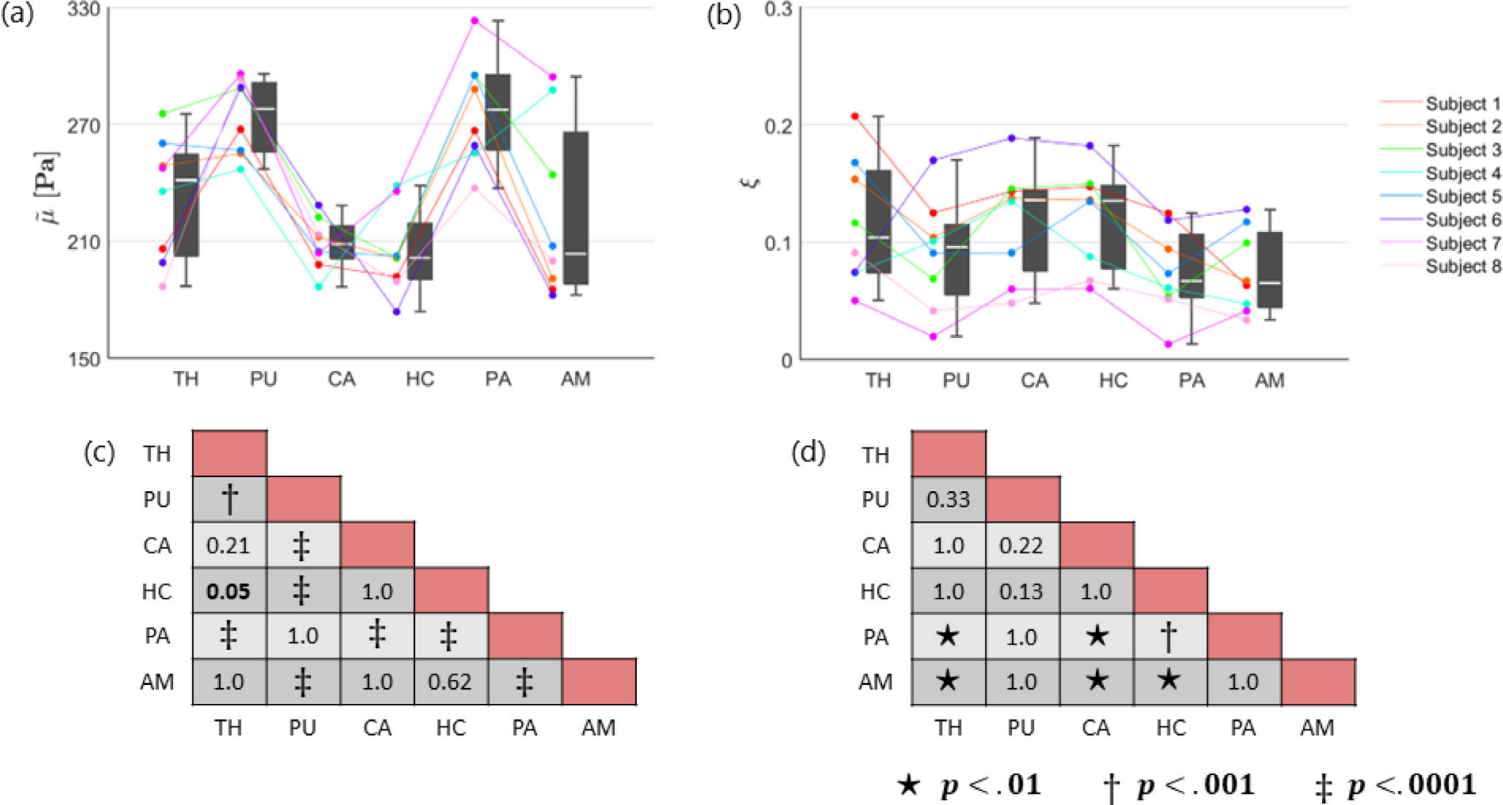
Boxplots alongside individual trend lines for each subject and significance charts in selected subcortical ROIs for (a) shear stiffness, $\tilde{\mu }$, and (b) damping ratio, ξ. Each dot represents the mean within one region for one subject. The regions are sorted by number of voxels such that the largest regions appear on the left. The white line within the boxplots correspond to the median and the upper and lower end of the boxplots represent the 25th and 75th quartiles. The whiskers illustrate the maximum and minimum values that are not outliers, where outliers are values which lie at least 1.5∙IQR away from the upper or lower quartiles. The significance charts for the (c) shear stiffness and (d) damping ratio were determined by utilizing a general linear mixed model and performing a post‐hoc analysis which was adapted for multiple comparisons using Bonferroni correction. Regions that are significantly different are indicated with bold text, and the symbols: ⋆, †, or ‡, depending on their level of significance. The shear stiffness has been denoted with a tilde to indicate the relative nature of the recovered solutions. The abbreviations for the regions can be found in the legend of Table 4.

The mean and standard deviation over all subjects within each subcortical GM region are provided in Table 4. The shear stiffness showed regional differences that were consistent between subjects, as visualized in plot (a) of Figure 6. Likewise, the damping ratio is presented in plot (b) of Figure 6 but exhibits overall less consistent regional differences between subjects. The pairwise comparisons of each ROI is presented as significance charts for (c) the shear stiffness and (d) the damping ratio.

**TABLE 4:** Mean and standard deviation of stiffness parameters in subcortical GM ROIs.

	Shear stiffness, $\tilde{\mu }$ (Pa)	Damping ratio, ξ
TH	232 ± 32	0.117 ± 0.055
PU	274 ± 20	0.090 ± 0.047
CA	208 ± 13	0.119 ± 0.048
HC	204 ± 22	0.121 ± 0.045
PA	277 ± 28	0.074 ± 0.037
AM	224 ± 46	0.075 ± 0.036

Abbreviations: AM, amygdala; CA, caudate; HC, hippocampus; PA, pallidum; PU, putamen; TH, thalamus.

### 3.3 Atlas comparison

**FIGURE 7:**
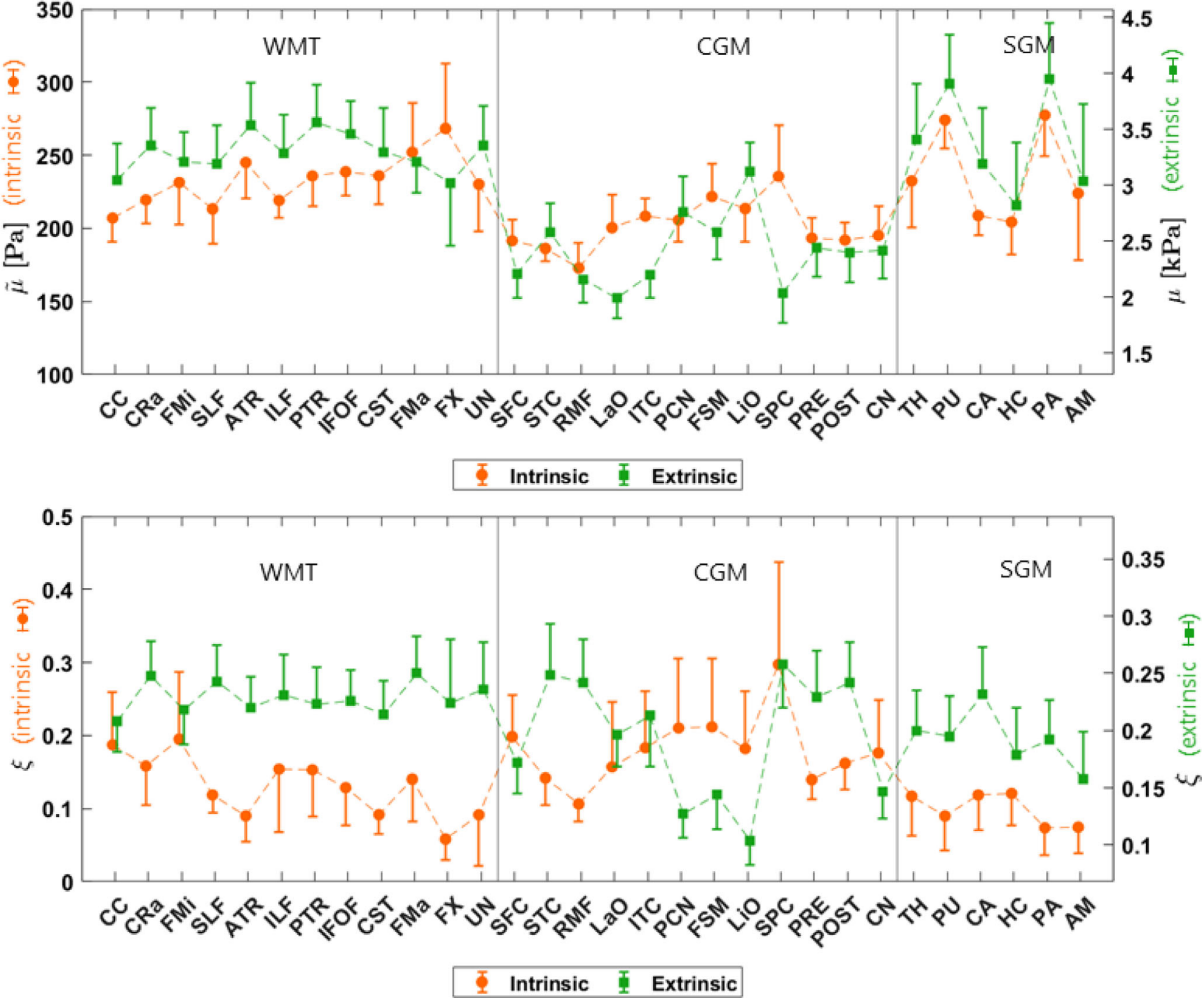
The mean shear stiffness, $\tilde{\mu }$ (top) and damping ratio, ξ (bottom) over all regions presented in this intrinsic analysis and the EVPASS. The whiskers represent the standard deviation in each ROI. The right‐hand axis is scaled using global mean values throughout the brain volume such that the relative values between the intrinsic and extrinsic analyses can be evaluated. The shear stiffness has been denoted with a tilde to the relative nature of the recovered solutions. The thin vertical lines were added as visual aid creating sections for WMTs, cortical gray matter (CGM) and subcortical gray matter (SGM). AM, amygdala; ATR, anterior thalamic radiation; CA, caudate; CC, corpus callosum; CN, cuneus; CRa, corona radiata; CST, corticospinal tract; FMa major forceps; FMi, minor forceps; FSM, fusiform gyrus; FX, fornix; HC, hippocampus; IFOF, inferior frontal‐occipital fasciculus; ILF, inferior longitudinal fasciculus; ITC, inferior temporal cortex; LaO, lateral occipital cortex; LiO, lingual occipital cortex; PA, pallidum; PCN, precuneus; POST, postcentral cortex; PTR, posterior thalamic radiation; PU, putamen; RMF, rostral middle frontal cortex; SFC, superior frontal cortex; SLF, superior longitudinal fasciculus; SPC, superior parietal cortex; STC, superior temporal cortex; TH, thalamus; UN, uncinated.

Figure 7 shows the mean shear stiffness and damping ratio in each ROI for the intrinsic data presented in this analysis in addition to the extrinsic data presented in the EVPASS. The intrinsic shear stiffness maps generally showed similar spatial patterns and regional differences as in the extrinsic case. The majority of disagreement is found in the cortical GM, with the superior parietal cortex exhibiting inverse behavior between the methods. For the damping ratio, the similarity of spatial patterns and regional differences were less evident, and many large differences between the methods can be observed. Spearman's rank correlation coefficient for all regions was calculated to be 0.74 for the shear stiffness and −0.13 for the damping ratio.

**FIGURE 8:**
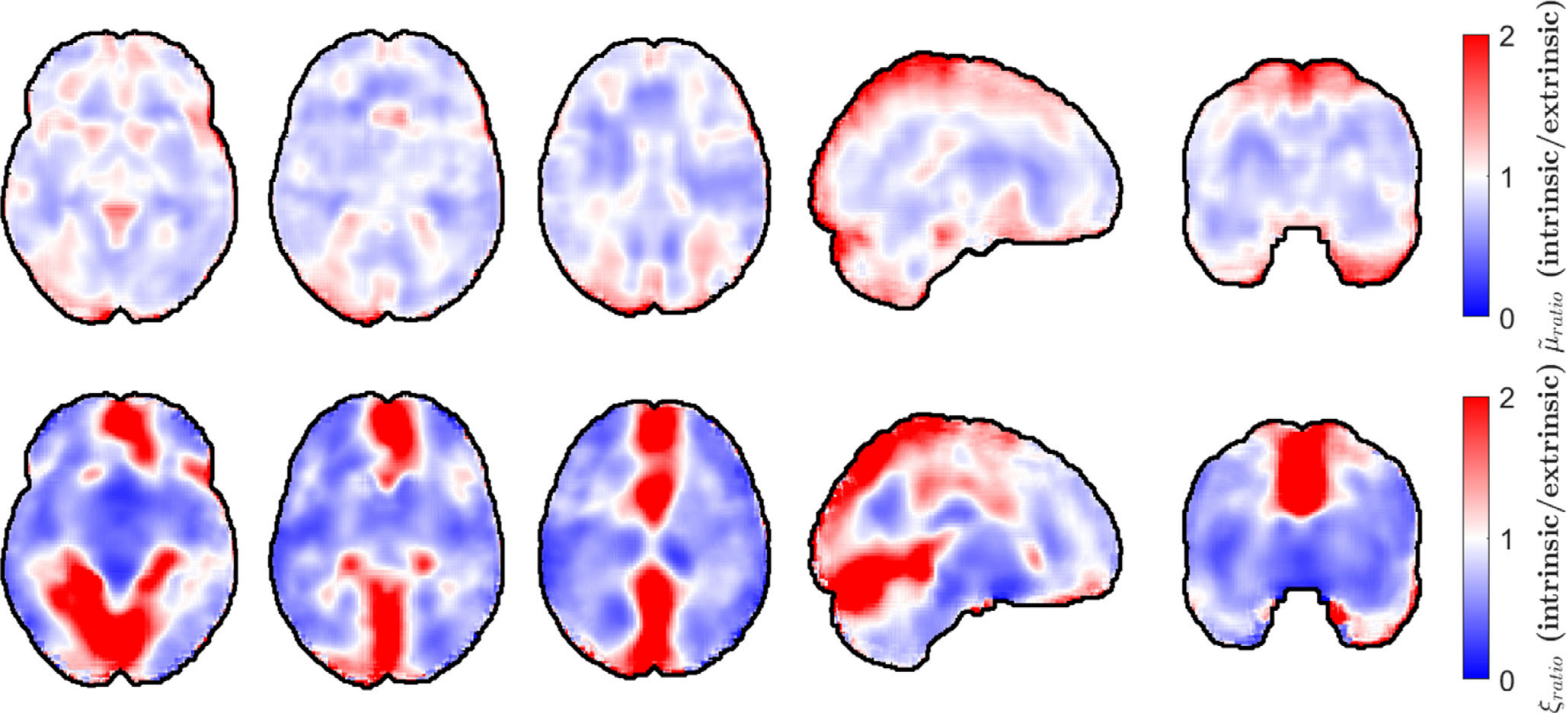
Transverse (a–c), sagittal (d), and coronal (e) slices of voxel‐wise shear stiffness difference maps of the mean distributions presented in this analysis and those presented in the EVPASS that is based on extrinsic actuation with 50 Hz (Hiscox et al., 2020). The respective slices are also shown for the damping ratio in (f–j). Each distribution is normalized by the global mean value throughout the brain volume. Red and blue areas correspond to regions of relatively higher and lower values respectively in the mean parameter maps obtained with intrinsic actuation.

The overall agreement in the shear stiffness and damping ratio maps is mapped in Figure 8, where the voxel‐wise ratios between the mean distributions reported in Figure 2 and the distributions presented in the EVPASS are presented. Here it is also evident that the majority of disagreement in the shear stiffness maps is located in the outer regions of the brain, especially toward the top and back of the brain. Some spatial patterns of disagreement can also be observed throughout the bulk of the brain. The damping ratio maps show extensive areas of significant disagreement in all shown slices, principally along the region of the falx cerebri and the separation of the two hemispheres. Some disagreement due to discretization effects from fluid flow masking can also be seen in slices (d, e) and (i, j) at the bottom of the brain.

### 3.4 Repeatability

**FIGURE 9:**
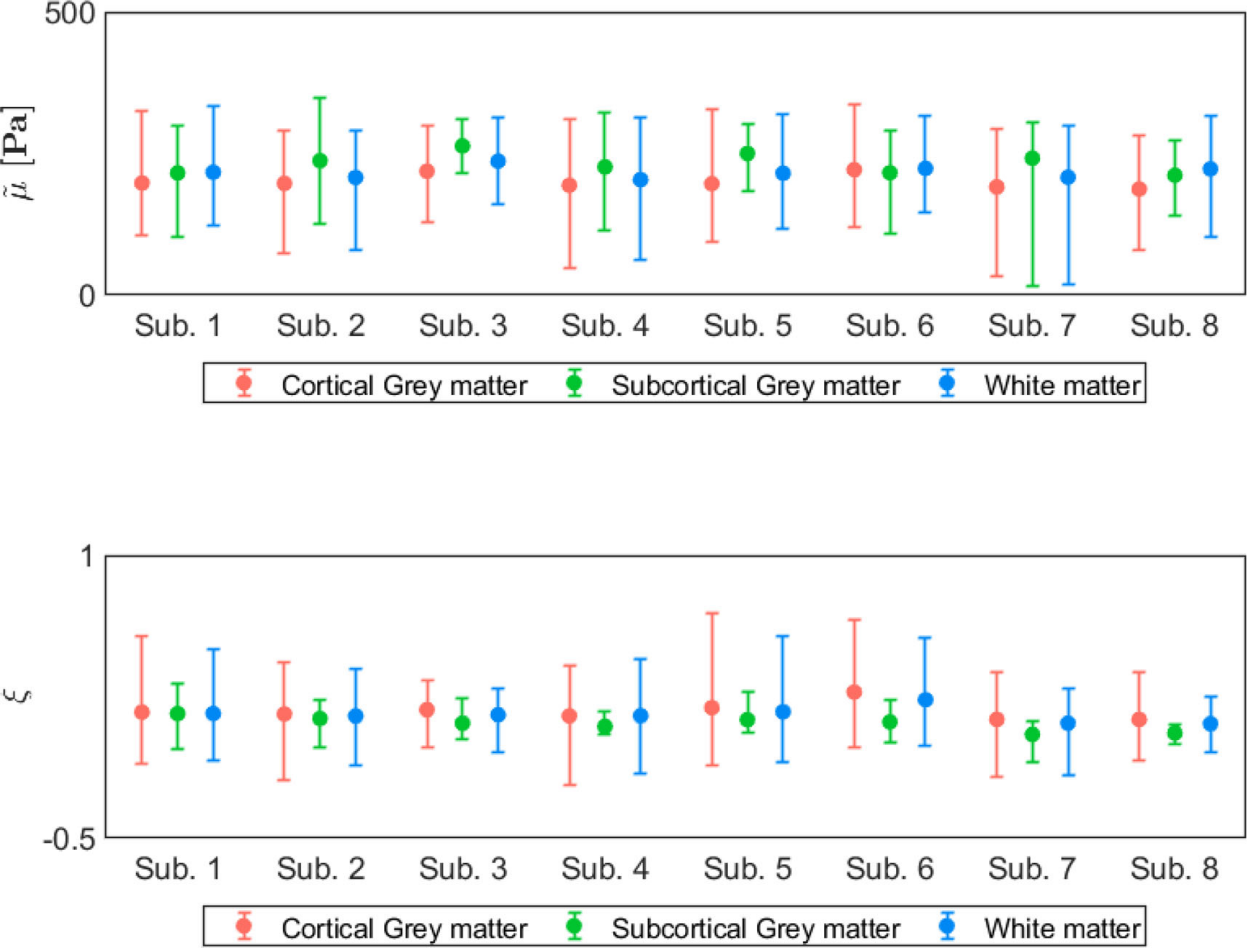
Summarized representation of a voxel‐wise analysis of the repeated measurements for the cortical GM, subcortical GM and WM in each subject. The error bars represent summarized Bland–Altman plots where the dot indicates the mean over all voxels for the two measurements. The whiskers represent the upper and lower limits of agreement, defined as b±1.96∙std where the bias b is the mean of the voxel‐wise differences between measurements. An asymmetry in length of the whiskers in one error bar reflects a difference in the mean between the repeated scans. Most error bars show symmetry in the length of the whiskers, suggesting significantly stable means between repeated measurements. Some variability is observed in the length of the whiskers between subjects, and probably reflects the difference in data quality due to variations in subject motion. The shear stiffness has been denoted with a tilde to the relative nature of the recovered solutions.

A summary of a comparison between the initial and repeated measurements is presented in Figure 9, which captures the representative features of the voxel‐wise Bland–Altman plots for each subject. Each analysis is done in the cortical GM, subcortical GM, and WM separately. Most of the error bars in Figure 9 show symmetry in the length of the whiskers, suggesting significantly stable means between repeated measurements. Some variability is observed in the length of the whiskers between subjects, and probably reflects the difference in data quality due to variation in subject motion. The full voxel‐wise Bland–Altman plots for all subjects are shown in Figures S3–S8

**FIGURE 10:**
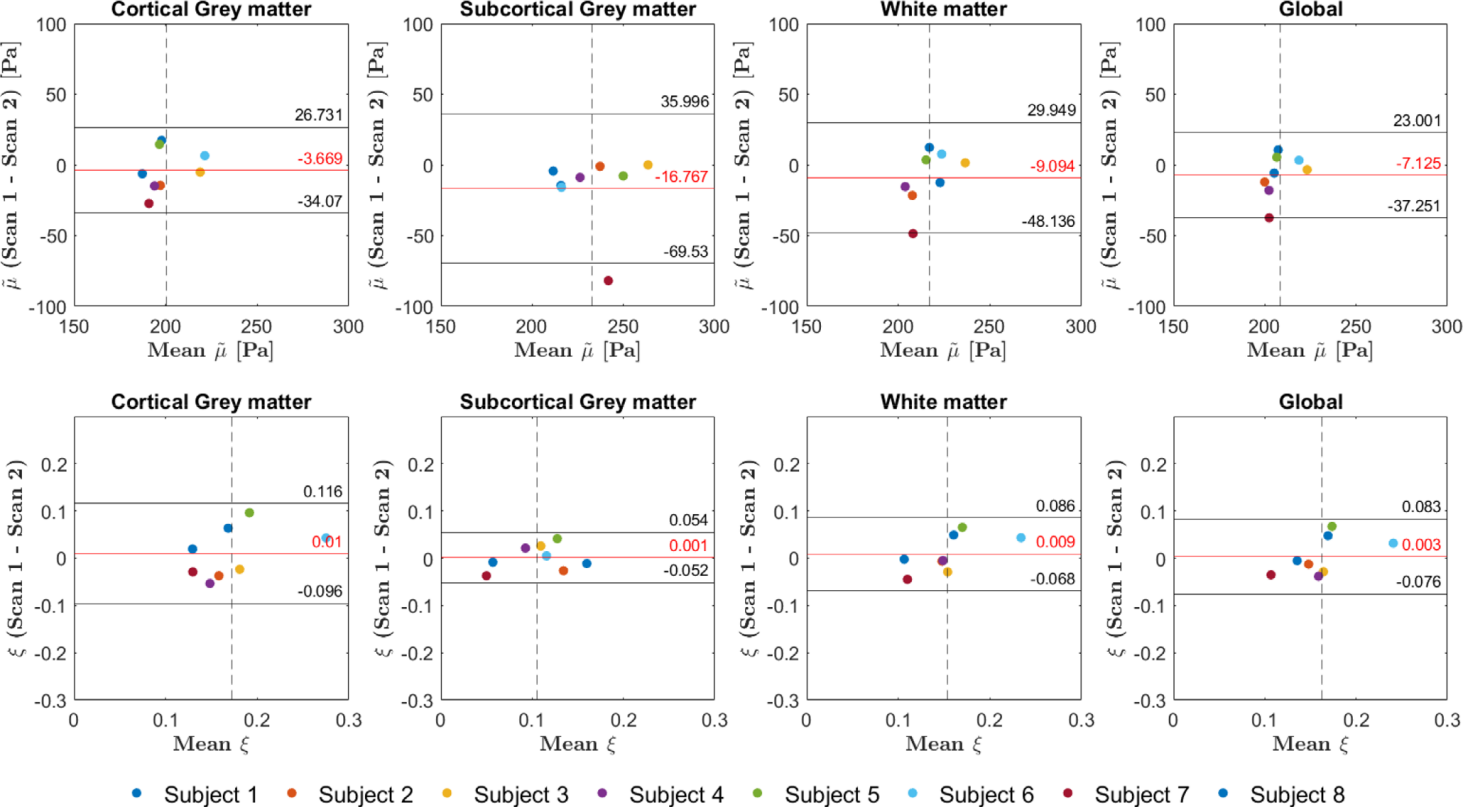
Bland–Altman plots of the mean shear stiffness (top row) and damping ratio (bottom row) in cortical GM, subcortical GM, WM, and global ROIs for all subjects. The horizontal red line represents the bias b which is the mean of the differences between the two measurements. The upper and lower horizontal black lines represent the upper and lower limits of agreement respectively, defined as b±1.96∙std. The vertical dashed line indicates the mean over all subjects.

Bland–Altman plots of the mean shear stiffness and damping ratio in the different global ROIs are presented in Figure 10 alongside the upper and lower limits of agreements. The root‐mean‐square error in shear stiffness was 15 Pa in cortical GM, 30.3 Pa in subcortical GM, 20.7 Pa in WM, and 16.0 in the whole brain. Similarly, the root‐mean‐square error in damping ratio was 0.051 in cortical GM and 0.025 in subcortical GM, 0.038 WM, and 0.038 in the whole brain for the damping ratio. The RC for shear stiffness was 18.4 ± 10.2 Pa in cortical GM, 23.3 ± 37.3 Pa in subcortical GM, 21.3 ± 20.6 Pa in WM, and 16.6 ± 15.8 Pa for the whole brain. For the damping ratio, the RC was 0.06 ± 0.04 in cortical GM, 0.03 ± 0.02 in subcortical GM, 0.04 ± 0.03 in WM, and 0.05 ± 0.03 for the whole brain. The median CV for shear stiffness was 5.2 ± 2.8% in cortical GM, 2.5 ± 7.9% in subcortical GM, 4.0 ± 5.1% in WM, and 2.8 ± 4.0% for the whole brain. For the damping ratio, the median CV was 16.2 ± 9.4% in cortical GM, 15.1 ± 15.6% in subcortical GM, 13.2 ± 11.1% in WM, and 14.6 ± 8.7% for the whole brain.

## 4 DISCUSSION

This work investigated the feasibility of utilizing intrinsic actuation‐MR elastography in the brain based on 7 T DENSE MRI measurements. The results demonstrated notable consistency between the viscoelastic property maps obtained from repeated displacement measurements. The shear stiffness maps showed fairly consistent spatial patterns. A voxel‐wise analysis performed separately in the grey and white matter demonstrated good repeatability of the method in both tissue types. Additionally, we assessed the ability of the method to detect normal regional variation in tissue stiffness, which potentially can serve as a proxy of expected differences between patients and controls. This was investigated by performing a regional analysis for different grey and WM ROIs, where various subregions within WMTs, cortical GM, and subcortical GM were distinguished based on their viscoelastic properties. Finally, we explored how results obtained with the intrinsic actuation approach related to those obtained with extrinsic actuation by directly comparing the measurements reported in this analysis with those presented in the EVPASS (Hiscox et al., 2020). The shear stiffness maps showed generally good agreement between the two methods while the damping ratio maps exhibited significantly more disagreement, as indicated by Spearman's rank correlation coefficient.

A significant increase of anatomical detail compared to previous attempts at intrinsic actuation‐based elastography could be seen in all presented stiffness maps (McGarry et al., 2019; Weaver et al., 2012; Zorgani et al., 2015), more than could be achieved by using higher field strength alone. Rather, the optimization of the acquisition sequence seemingly has a noticeable effect on the sensitivity. The first attempt at intrinsic MRE (Weaver et al., 2012) used a multiphase phase contrast gradient echo sequence in a 3 T scanner which allowed for a maximum sensitivity of 2.5 cm/s. Since then, there has only been two additional attempts at reconstructing mechanical property maps using the intrinsic MRE method (McGarry et al., 2019; Zorgani et al., 2015). Zorgani et al. employed a single‐shot echo planar gradient echo sequence in a 1.5 T scanner and used seismic noise correlation to characterize the tissue mechanics, resulting in a sensitivity slightly above 1 cm/s. McGarry et al. used the Philips QFLOW sequence in a 3 T scanner with a sensitivity of 2.5 cm/s, leading to a considerable increase in detail in the shear stiffness maps compared to the initial two attempts. Although only one shear stiffness map was presented by McGarry et al., its quality suggests that intrinsic MRE could also be viable on 3 T scanners. In contrast to the lower sensitivities of the previous intrinsic MRE attempts, the DENSE sequence in its current implementation allows for a sensitivity of around 1 mm/s, which implies almost an order of magnitude higher SNR and helps explain the vast improvement of detail in the parameter maps. The lack of further exploration of the intrinsic elastography method can probably be attributed to the significant noise seen in the parameter maps of the initial two attempts. The demonstration of the stability of the parameter maps between repeated measurements as presented in this work might therefore renew interest in intrinsic elastography.

The shear stiffness maps were found to be generally symmetric, with the average difference between symmetric brain regions being 0.2% for the shear stiffness and 6.5% for the damping ratio. Asymmetries observed in these results in regions that show good agreement across the repeated images are most likely due to data‐model mismatch between the measured displacement field and the purely viscoelastic model. This is due to the viscoelastic model not being able to provide a mechanism for relative fluid‐tissue movement. In regions where such fluid–tissue interaction is significant, the optimization‐based reconstruction process will determine effective parameter values which provide the best possible interpretation of this phenomenon in the context of the viscoelastic parameters, that is, either effective stiffness or effective damping effects. A poroelastic brain model, such as those presented in previous work for MRE with extrinsic activation (McGarry et al., 2015, 2019; Perriñez et al., 2010; Sowinski et al., 2021), would be able to correctly account for this relative fluid‐tissue movement and thus reduce such asymmetries.

We found that the shear stiffness is on average 9% higher in WM compared to cortical GM, which is in line with multiple previous studies (Braun et al., 2014; Hiscox et al., 2020; Johnson, McGarry, Gharibans, et al., 2013; Weickenmeier et al., 2016), that consistently report higher shear stiffness in WM compared to GM. Despite the fact that there is general agreement that WM is stiffer, the ratio between grey and white matter stiffness varies substantially across studies. This could be explained by the fact that the shear stiffness tends to exhibit considerable regional variation across the entire brain as reported before (Budday et al., 2020; Hiscox et al., 2020). Such regional variability tends to be much larger than the average difference between global GM and WM, which implies that one WM‐or GM value is a poor metric in this case. This effect can also be observed in our results, where the shear stiffness can vary by up to 60% (pallidum and rostral middle frontal cortex) between various ROIs. There are multiple factors that could contribute to such stiffness variability. Not only is the mechanical structure of the brain likely adapted to regionally varying functional demands (Budday et al., 2020), but also fluid‐based effects like perfusion probably contribute to the tissue stiffness (Hetzer et al., 2018). Variability in stiffness parameters between different brain regions is clearly observed in the cortical GM, the subcortical GM, and the WMT. Most subjects follow similar trends in regional shear stiffness with occasional outliers, likely due to *hotspot*‐like artifacts that tend to appear sporadically throughout the property maps. The intra‐subject variance tends to increase for the smallest regions, which is particularly clear for the uncinate and fornix, which are on average 59 and 92 voxels in size, respectively. Additionally, the variance seems to increase for regions in close vicinity to the ventricles, such as the minor forceps, possibly due to remaining partial volume effects with CSF.

The relative shear stiffness between WMTs shows generally high correspondence to the EVPASS. As is observed in the atlas, the vast majority of investigated WMTs exhibit higher shear stiffness than the global WM (217 ± 15 Pa), with the exceptions being the superior longitudinal fasciculus and the corpus callosum. We also observe the corona radiate being stiffer than the corpus callosum, which is in line with multiple previous studies (Budday et al., 2017; Hiscox et al., 2020; Johnson, McGarry, Gharibans, et al., 2013). We observe very similar regional trends for the shear stiffness in the subcortical GM with those found in extrinsic actuation. The pallidum and putamen are found to have the highest shear stiffness in the subcortical GM, something that has been observed across multiple MRE studies (Hetzer et al., 2018; Hiscox et al., 2020; Johnson et al., 2016). The relative regional shear stiffness in the cortical GM shows generally lower correspondence to the equivalent regions presented in the EVPASS. For example, the superior parietal cortex displays an inverted behavior, where it exhibits the largest shear stiffness in our results and second to lowest stiffness in the EVPASS. There are multiple factors that might play a role in these discrepancies, the first of which being that the low‐resolution finite element mesh implemented in NLI treats the complex geometry exhibited due to gyri and sulci in the cortical GM as a single continuum (Hiscox et al., 2020). As discussed above, the periphery of the brain is also the area of largest uncertainty, as fluid flow due to CSF might introduce artifacts despite a significant portion of the CSF being masked. Additionally, the application of fluid flow masks might itself introduce some uncertainty as it affects the geometry of the finite element models used in NLI due to certain nodes being excluded.

The voxel‐wise difference distributions shown in Figure 8 reveal some larger regions of disagreement between intrinsic and extrinsic actuation. The most pronounced differences are along the periphery of the brain, particularly towards the top back of the brain. Part of the reason might be that each subject is masked prior to inversion and then registered to standard space in both our work and in the EVPASS. The edges of the property maps therefore become somewhat different between subjects, leading to increased uncertainty. Furthermore, such regions tend to contain larger amounts of CSF and larger blood vessels. The differences might therefore be an effect of how fluid and fluid–tissue partial volume effects get treated differently in the two approaches. Some inherent variance also follows from the relatively limited sample size used in this analysis particularly also the existence of hotspots might bias certain regions towards higher shear stiffness. It is also possible that the stiffening effect of the brain under the common actuation frequencies used in extrinsic MRE may have a regional dependence due to the heterogeneous mechanical structure of the brain. To the authors' knowledge, this effect has not yet been examined and could be a subject for future investigation. As can be seen in Figure 7, there seems to be in general less variability in shear stiffness across brain structures when using intrinsic MRE, potentially due to this effect. It could, however, also be a result of limited regional sensitivity of intrinsic MRE.

The voxel‐wise Bland–Altman analysis further establishes the repeatability of intrinsic‐based elastography. There is certain dissimilarity in some measurements, which is likely to mainly due to the hotspots. This is particularly clear in Subject 7, where hotspots span large parts of the center of the shear stiffness map in the repeated scan (Figure S1) and is consequently reflected in the length of the bottom whisker in the summary plot of the voxel‐wise Bland–Altman analysis (Figure 9). Such hotspots consequently have an effect on the statistics, resulting in large standard deviations in the root‐mean‐square error, the RC and the CV. Although a large portion of hotspots were successfully removed by masking high‐displacement voxels in the displacement data induced by fluid flow, a considerable number still remained of which the origin is uncertain. Indeed, there are many areas within the bulk of the brain that contain additional CSF structures and larger blood vessels, which our current heuristic filtering method ignored as masking such voxels would result in gaps in the finite element model mesh. Other possibilities of the origin of hotspots include artifacts in displacement data due to motion‐induced phase inconsistencies in the multishot (segmented) 3D EPI readout scheme (Adams et al., 2020; Soellinger et al., 2009) or data‐model mismatch. Noisy data could also be a source of hotspots, but that is less likely given the high SNR of the motion maps with the used 7 T imaging method.

Despite the uncertainty introduced by hotspots, our method shows comparable repeatability to that of extrinsic MRE, as reported by Svensson et al. (2021). They used a 3 T scanner and a gradient echo sequence with a vibration frequency of 50 Hz to image the brains of 15 healthy subjects. Using FEM reconstruction, they report a whole‐brain RC of 9.6 ± 2.3% relative to the mean whole‐brain shear stiffness, and an upper and lower limit of agreement of 9.6% and 6.2% respectively. We report a whole‐brain RC of 8.0 ± 7.6% relative to the mean whole‐brain stiffness, and an upper and lower limit of agreement of 11.0% and 17.8% respectively. Both the RCs and the upper limits of agreement show good agreement while the lower limit of agreement presented here is considerably larger, which can be largely attributed to the hotspots present in the second scan of Subject 7. Svensson et al. along with other groups has also looked into the CV as a metric of repeatability. In our case, CV measurements are of limited value given that we only have two repeats per subject, but the whole‐brain median CV for shear stiffness across all subjects of 2.7% still compares well with the values ranging from 0.67% to 4.1% reported in literature (Herthum et al., 2022; Huang et al., 2019; Johnson et al., 2016; Murphy et al., 2013; Svensson et al., 2021).

In contrast to the shear stiffness distributions repeatedly showing consistent brain structure and general agreement with the EVPASS, the damping ratio distributions display a more inconsistent, ambiguous structure. This is especially apparent when comparing the RC between the two quantities, where it was 8% for the shear stiffness and 28% for the damping ratio when expressed as a percentage of the global shear stiffness and damping ratio respectively. These inconsistencies can in part be explained by the fact that the damping ratio is composed of two reconstructed parameters, $G^{\prime }$ and *G*″, which yields unfavorable noise propagation from both parameters. Moreover, data‐model mismatch could also affect the damping ratio when trying to model the brain under low frequencies as a viscoelastic material. As been discussed by McGarry et al. (2015, 2019), the choice of material model is significantly dependent on the actuation frequency, where poroelastic effects are more important under the low frequencies used in intrinsic actuation. Instead, the simpler viscoelastic model is more appropriate for the higher frequencies of extrinsic actuation, where the poroelastic effects diminish. It is thus plausible that the inconsistencies observed in the damping ratio are due to data‐model mismatch, as it is a purely viscoelastic quantity. Shifting towards the use of a poroelastic brain model in intrinsic MRE may be the next step in resolving such inconsistencies (Budday et al., 2020; Herthum et al., 2021; Hiscox et al., 2020; Johnson, McGarry, Van Houten, et al., 2013; McGarry et al., 2019; Zhang et al., 2011).

Although the performance of the intrinsic MRE approach looks promising on many levels, there are some limitations. The occurrence of hotspots throughout the stiffness parameter distributions introduces inconsistency across the measurements. However, recent developments in the optimization of the DENSE sequence (Sloots et al., 2021) might offer significant improvement in not only general noise, but also in sensitivity to brain tissue motion. This could significantly help diminish the number of artifacts that arise due to fluid flow. Such improvement to the DENSE sequence involves measuring deformation gradients rather than displacements of the tissue, which requires adapting the NLI scheme to account for deformation measurements. In this work, we see potential shortcomings when modeling the brain as a viscoelastic material under low frequencies in mainly two ways: the recovery of non‐unique solutions and the relative inconsistent characterization of the damping ratio distribution. As mentioned before, implementing a poroelastic model could potentially help alleviate both problems, while introducing different challenges. For instance, poroelastography methods are highly sensitive to the prescribed hydraulic permeability values at low frequencies (McGarry et al., 2019), which necessitates accurate measurements or estimations of these values. Future work aims to implement both an improved DENSE sequence in the acquisition and the poroelastic model in the NLI scheme to further explore the potential of intrinsic actuation MRE.

## 5 CONCLUSION

In this study, 7 T MR displacement measurements of cardiac‐induced brain tissue deformation were used to successfully reconstruct the viscoelastic properties of the brain in eight healthy subjects. The sensitivity of the method for detecting differences in stiffness was also explored by performing a region‐wise analysis where the viscoelastic properties could consistently be distinguished between various brain regions. The repeatability was investigated using repeated scans and was found to be generally consistent. The major challenges of the current implementation include hotspot‐like artifacts due to fluid flow and the recovery of non‐unique viscoelastic solutions at low frequencies. Future work thus aims to investigate the performance of adapting the NLI scheme for deformation measurements and poroelastic reconstruction to deal with such challenges.

## FUNDING INFORMATION

This work was supported by a Vici Grant from the Netherlands Organization for Scientific Research (NWO) awarded to Jaco J. M. Zwanenburg under grant agreement no. 18674, and by the European Research Council under the European Union's Seventh Framework Programme (FP7/2007–2013)/ERC grant agreement no. 337333.

## Supporting information


**FIGURE S1:** Representative axial slices of the shear stiffness, $\tilde{\mu }$, for all subjects alongside the repeated scans which have been rigidly registered to the original scans. Each repeated scan shows significant structural similarity to the original scan, with the exception of large‐valued hotspots that likely arise due to fluid flow (exampled are indicated with red arrow). A more significant hotspot (indicated with blue arrow) can be seen in scan 2 of Subject 7, likely due to subject motion in the scanner.
**FIGURE S2:** Representative axial slices of the damping ratio, ξ, for all subjects alongside the repeated scans which have been rigidly registered to the original scans.
**FIGURE S3:** Bland–Altman plots for the WM shear stiffness in all subjects. The horizontal red line represents the bias b which is the mean of the voxel‐wise differences between the two measurements. The upper and lower horizontal black lines represent the upper and lower limits of agreement respectively, defined as b±1.96∙std. The vertical dashed line indicates the mean over all voxels for the two measurements.
**FIGURE S4:** Bland–Altman plots for the WM damping ratio in all subjects. The horizontal red line represents the bias b which is the mean of the voxel‐wise differences between the two measurements. The upper and lower horizontal black lines represent the upper and lower limits of agreement respectively, defined as b±1.96∙std. The vertical dashed line indicates the mean over all voxels for the two measurements.
**FIGURE S5:** Bland–Altman plots for the cortical GM shear stiffness in all subjects. The horizontal red line represents the bias b which is the mean of the voxel‐wise differences between the two measurements. The upper and lower horizontal black lines represent the upper and lower limits of agreement respectively, defined as b±1.96∙std. The vertical dashed line indicates the mean over all voxels for the two measurements.
**FIGURE S6:** Bland–Altman plots for the cortical GM damping ratio in all subjects. The horizontal red line represents the bias b which is the mean of the voxel‐wise differences between the two measurements. The upper and lower horizontal black lines represent the upper and lower limits of agreement respectively, defined as b±1.96∙std. The vertical dashed line indicates the mean over all voxels for the two measurements.
**FIGURE S7:** Bland–Altman plots for the subcortical GM shear stiffness in all subjects. The horizontal red line represents the bias b which is the mean of the voxel‐wise differences between the two measurements. The upper and lower horizontal black lines represent the upper and lower limits of agreement respectively, defined as b±1.96∙std. The vertical dashed line indicates the mean over all voxels for the two measurements.
**FIGURE S8:** Bland–Altman plots for the subcortical GM damping ratio in all subjects. The horizontal red line represents the bias b which is the mean of the voxel‐wise differences between the two measurements. The upper and lower horizontal black lines represent the upper and lower limits of agreement respectively, defined as b±1.96∙std. The vertical dashed line indicates the mean over all voxels for the two measurements.
**TABLE S1:** Number of voxels in each white matter tract ROIs in 2 mm isotropic resolution.
**TABLE S2:** Number of voxels in each cortical GM ROIs in 2 mm isotropic resolution.
**TABLE S3:** Number of voxels in each subcortical GM ROIs in 2 mm isotropic resolution.

## Data Availability

The data that support the findings of this study are available from the corresponding author upon reasonable request.
