# Correction to “Estimating the Viscoelastic Properties of the Human Brain at 7 T MRI Using Intrinsic MRE and Nonlinear Inversion”

**DOI:** 10.1002/hbm.70117

**Published:** 2024-12-20

Burman Ingeberg, M., E. Van Houten, and J. J. M. Zwanenburg. 2023. “Estimating the Viscoelastic Properties of the Human Brain at 7 T MRI Using Intrinsic MRE and Nonlinear Inversion.” *Human Brain Mapping* 44, no. 18: 6575–6591. https://doi.org/10.1002/hbm.26524.

In caption of Table 1, the text “WMT ROIs” should be replaced by “global ROIs”.

In the body of Table 1, the shear stiffness values in subcortical GM of 213 ± 21 should be replaced with 233 ± 24.

In the abbreviations of Table 3, the abbreviation of the precentral cortex is missing. Thus, “PRE, precentral cortex;” should be added after “POST, postcentral cortex;”

In paragraph 2 of the “3.4 Repeatability” section, the repeatability coefficients (RC) were incorrectly calculated. Thus, the text

“The RC for shear stiffness was 18.4 ± 10.2 Pa in cortical GM, 23.3 ± 37.3 Pa in subcortical GM, 21.3 ± 20.6 Pa in WM, and 16.6 ± 15.8 Pa for the whole brain. For the damping ratio, the RC was 0.06 ± 0.04 in cortical GM, 0.03 ± 0.02 in subcortical GM, 0.04 ± 0.03 in WM, and 0.05 ± 0.03 for the whole brain.”

Should read

“The RC for shear stiffness was 29.3 Pa in cortical GM, 59.3 Pa in subcortical GM, 40.6 Pa in WM, and 31.4 Pa for the whole brain. For the damping ratio, the RC was 0.10 in cortical GM, 0.05 in subcortical GM, 0.07 in WM, and 0.07 for the whole brain.”

Due to the incorrect calculation of the repeatability coefficient, the following sections also contain errors.

In paragraph 8 of the “Discussion” section, the text

“We report a whole‐brain RC of 8.0% ± 7.6% relative to the mean whole‐brain stiffness, and an upper and lower limit of agreement of 11.0% and 17.8% respectively. Both the RCs and the upper limits of agreement show good agreement while the lower limit of agreement presented here is considerably larger, which can be largely attributed to the hotspots present in the second scan of Subject 7.”

Should read

“We report a whole‐brain RC of 15.1% relative to the mean whole‐brain stiffness, and an upper and lower limit of agreement of 11.0% and 17.8% respectively. Both the RCs and the upper limits of agreement show relatively good agreement, given the difference in number of subjects. The lower limit of agreement presented here is considerably larger, which can be largely attributed to the hotspots present in the second scan of Subject 7.”

In paragraph 9 of the “Discussion” section, the text

“This is especially apparent when comparing the RC between the two quantities, where it was 8% for the shear stiffness and 28% for the damping ratio when expressed as a percentage of the global shear stiffness and damping ratio respectively.”

Should read

“This is especially apparent when comparing the RC between the two quantities, where it was 15.1% for the shear stiffness and 45.9% for the damping ratio when expressed as a percentage of the global shear stiffness and damping ratio respectively.”

In the abstract, the text

“The whole‐brain repeatability coefficient (RC) for shear stiffness was (mean ± standard deviation) 8% ± 8% relative to the mean whole‐brain stiffness, and the damping ratio RC was 28% ± 17% relative to the whole‐brain damping ratio.”

Should read

“The whole‐brain repeatability coefficient (RC) for shear stiffness was 15.1% relative to the mean whole‐brain stiffness, and the damping ratio RC was 45.9% relative to the whole‐brain damping ratio.”

We apologize for these mistakes.

