# Supplementary material for: Estimating the viscoelastic properties of the human brain at 7 T MRI using intrinsic MRE and nonlinear inversion
Source: Hum Brain Mapp. 2023 Nov 1;44(18):6575–91. doi: 10.1002/hbm.26524 (PMC10681656; doi:10.1002/hbm.26524)
Supplement: Supplementary file 1 — FIGURE S1: Representative axial slices of the shear stiffness, μ~, for all subjects alongside the repeated scans which have been rigidly registered to the original scans. Each repeated scan shows significant structural similarity to the original scan, with the exception of large‐valued hotspots that likely arise due to fluid flow (exampled are indicated with red arrow). A more significant hotspot (indicated with blue arrow) can be seen in scan 2 of Subject 7, likely due to subject motion in the scanner. FIGURE S2: Representative axial slices of the damping ratio, ξ, for all subjects alongside the repeated scans which have been rigidly registered to the original scans. FIGURE S3: Bland–Altman plots for the WM shear stiffness in all subjects. The horizontal red line represents the bias b which is the mean of the voxel‐wise differences between the two measurements. The upper and lower horizontal black lines represent the upper and lower limits of agreement respectively, defined as b±1.96∙std. The vertical dashed line indicates the mean over all voxels for the two measurements. FIGURE S4: Bland–Altman plots for the WM damping ratio in all subjects. The horizontal red line represents the bias b which is the mean of the voxel‐wise differences between the two measurements. The upper and lower horizontal black lines represent the upper and lower limits of agreement respectively, defined as b±1.96∙std. The vertical dashed line indicates the mean over all voxels for the two measurements. FIGURE S5: Bland–Altman plots for the cortical GM shear stiffness in all subjects. The horizontal red line represents the bias b which is the mean of the voxel‐wise differences between the two measurements. The upper and lower horizontal black lines represent the upper and lower limits of agreement respectively, defined as b±1.96∙std. The vertical dashed line indicates the mean over all voxels for the two measurements. FIGURE S6: Bland–Altman plots for the cortical GM damping ratio in all sub [file HBM-44-6575-s001.docx]

# Supplementary material


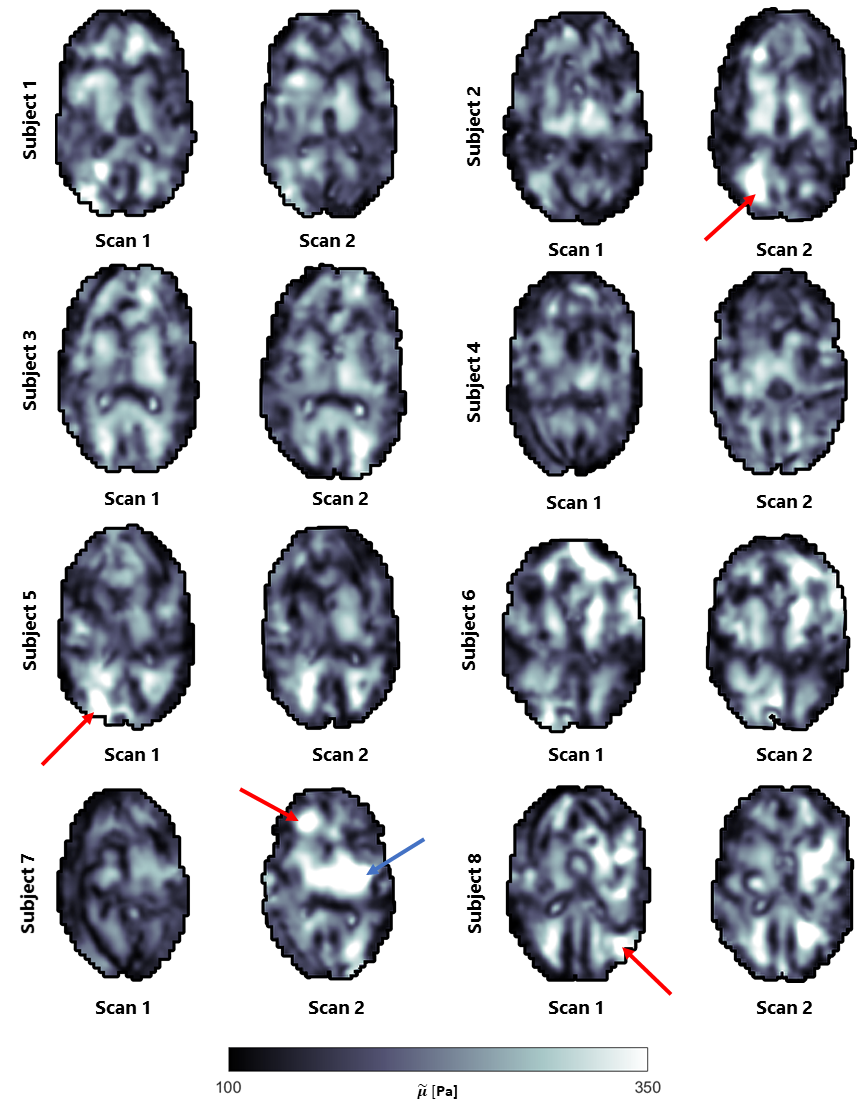


Figure S1: Representative axial slices of the shear stiffness, $\tilde{\mu}$, for all subjects alongside the repeated scans which have been rigidly registered to the original scans. Each repeated scan shows significant structural similarity to the original scan, with the exception of large-valued hotspots that likely arise due to fluid flow (exampled are indicated with red arrow). A more significant hotspot (indicated with blue arrow) can be seen in scan 2 of Subject 7, likely due to subject motion in the scanner.


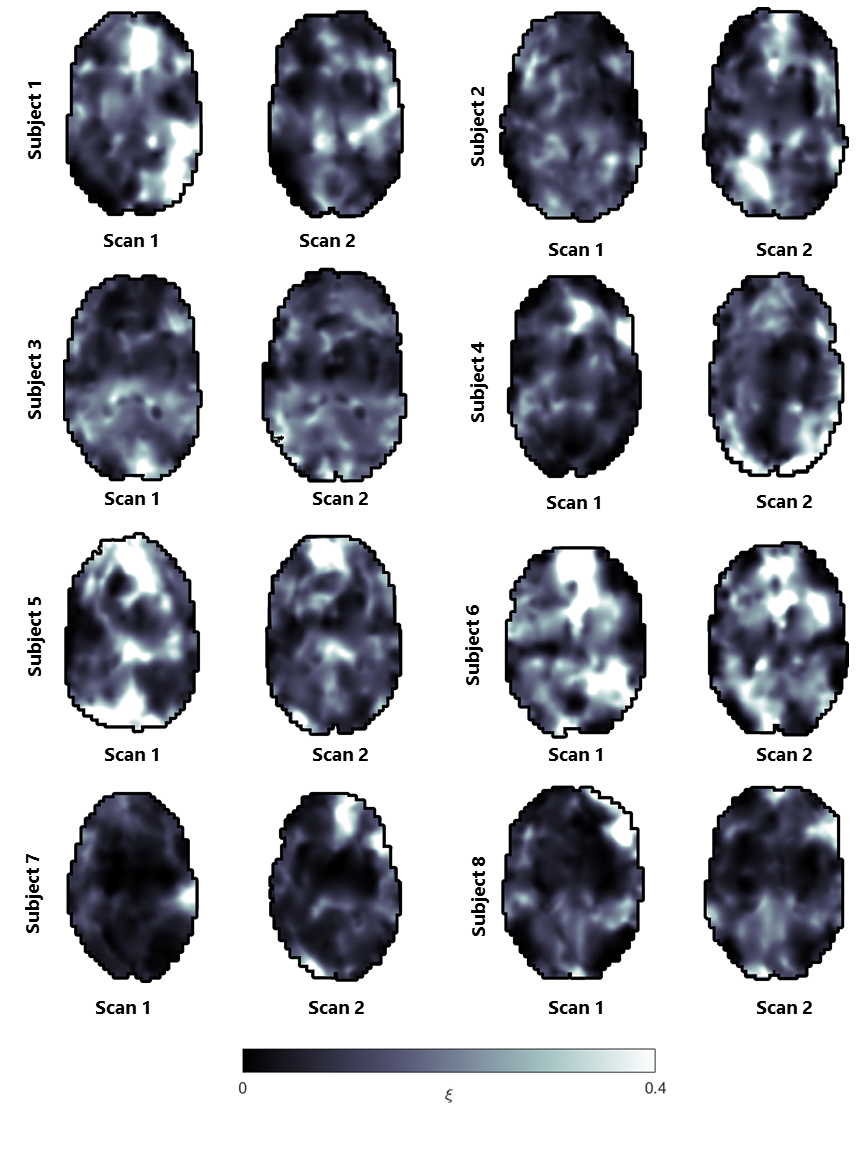
Figure S2: Representative axial slices of the damping ratio, $\xi$, for all subjects alongside the repeated scans which have been rigidly registered to the original scans.


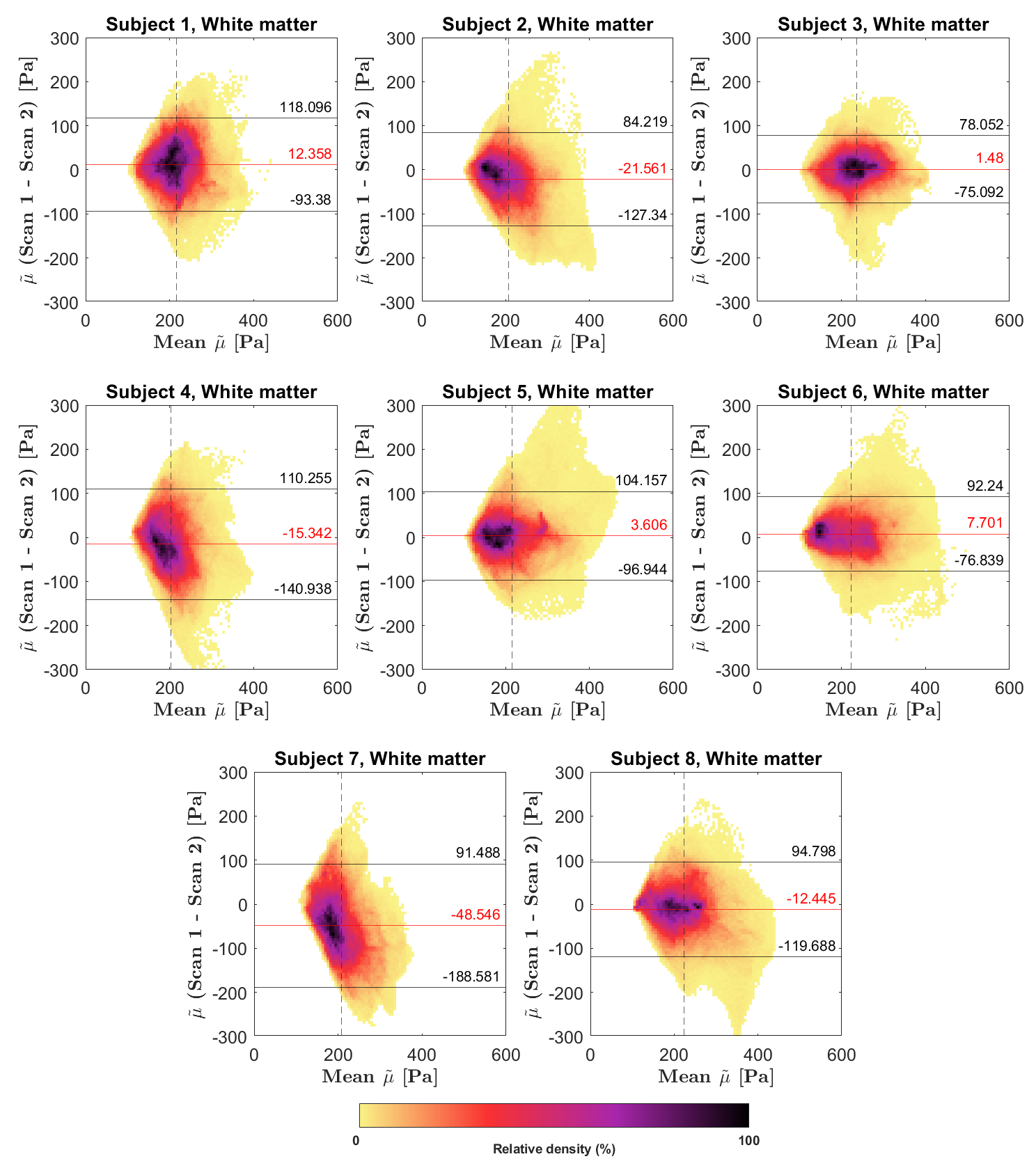


Figure S3: Bland-Altman plots for the WM shear stiffness in all subjects. The horizontal red line represents the bias $b$ which is the mean of the voxel-wise differences between the two measurements. The upper and lower horizontal black lines represent the upper and lower limits of agreement respectively, defined as $b\pm1.96\cdot std$. The vertical dashed line indicates the mean over all voxels for the two measurements.


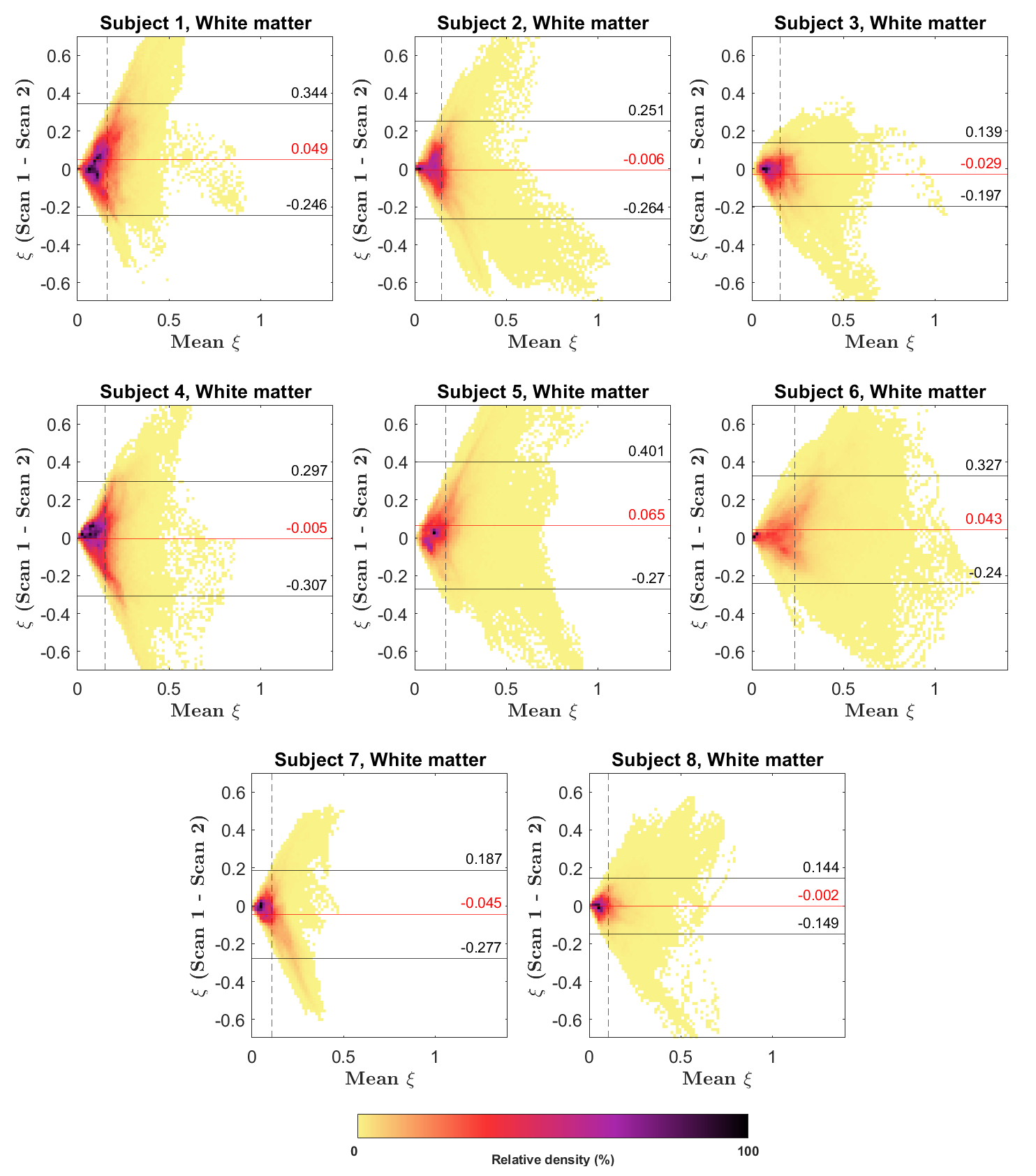


Figure S4: Bland-Altman plots for the WM damping ratio in all subjects. The horizontal red line represents the bias $b$ which is the mean of the voxel-wise differences between the two measurements. The upper and lower horizontal black lines represent the upper and lower limits of agreement respectively, defined as $b\pm1.96\cdot std$. The vertical dashed line indicates the mean over all voxels for the two measurements.


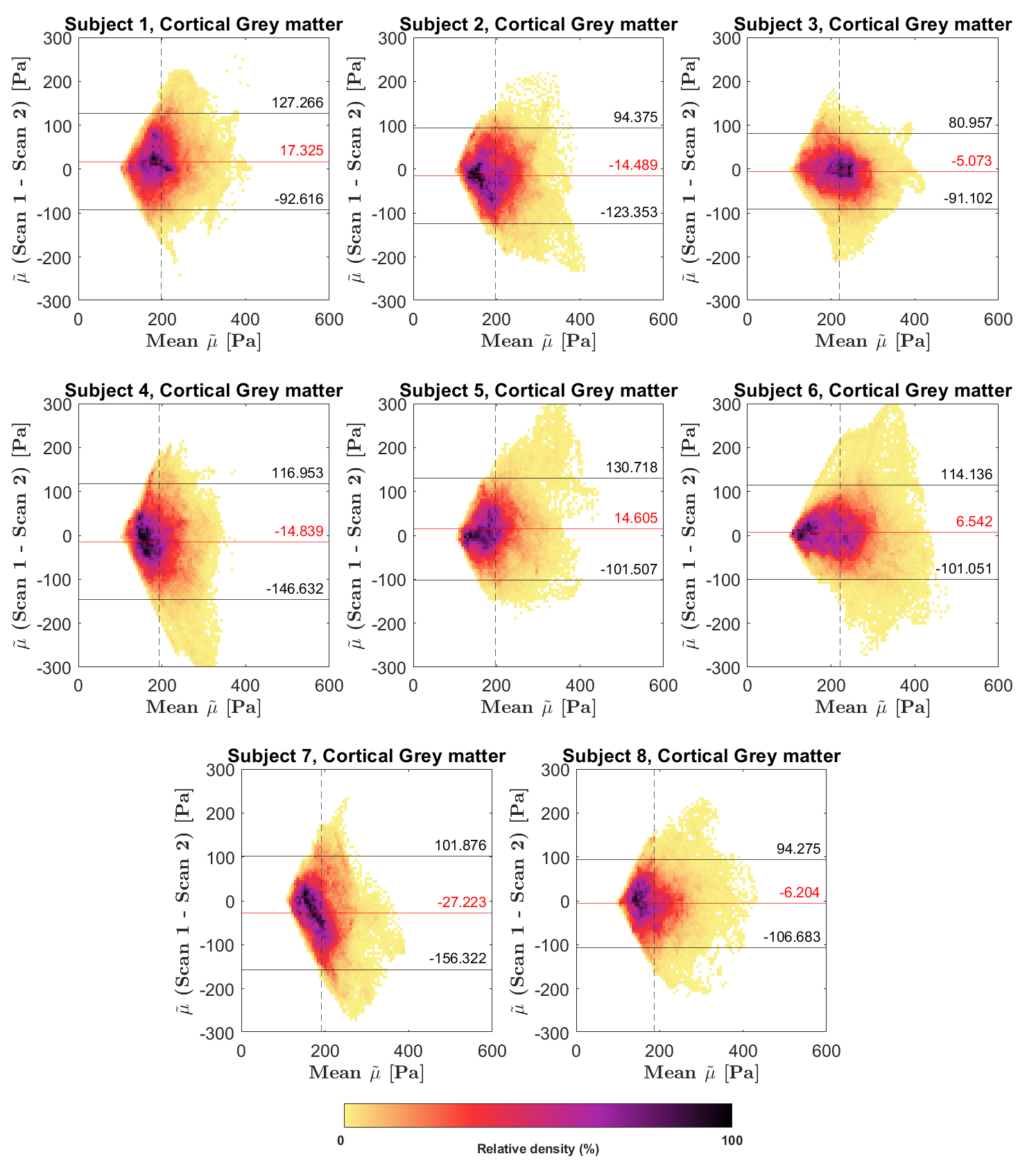


Figure S5: Bland-Altman plots for the cortical GM shear stiffness in all subjects. The horizontal red line represents the bias $b$ which is the mean of the voxel-wise differences between the two measurements. The upper and lower horizontal black lines represent the upper and lower limits of agreement respectively, defined as $b\pm1.96\cdot std$. The vertical dashed line indicates the mean over all voxels for the two measurements.


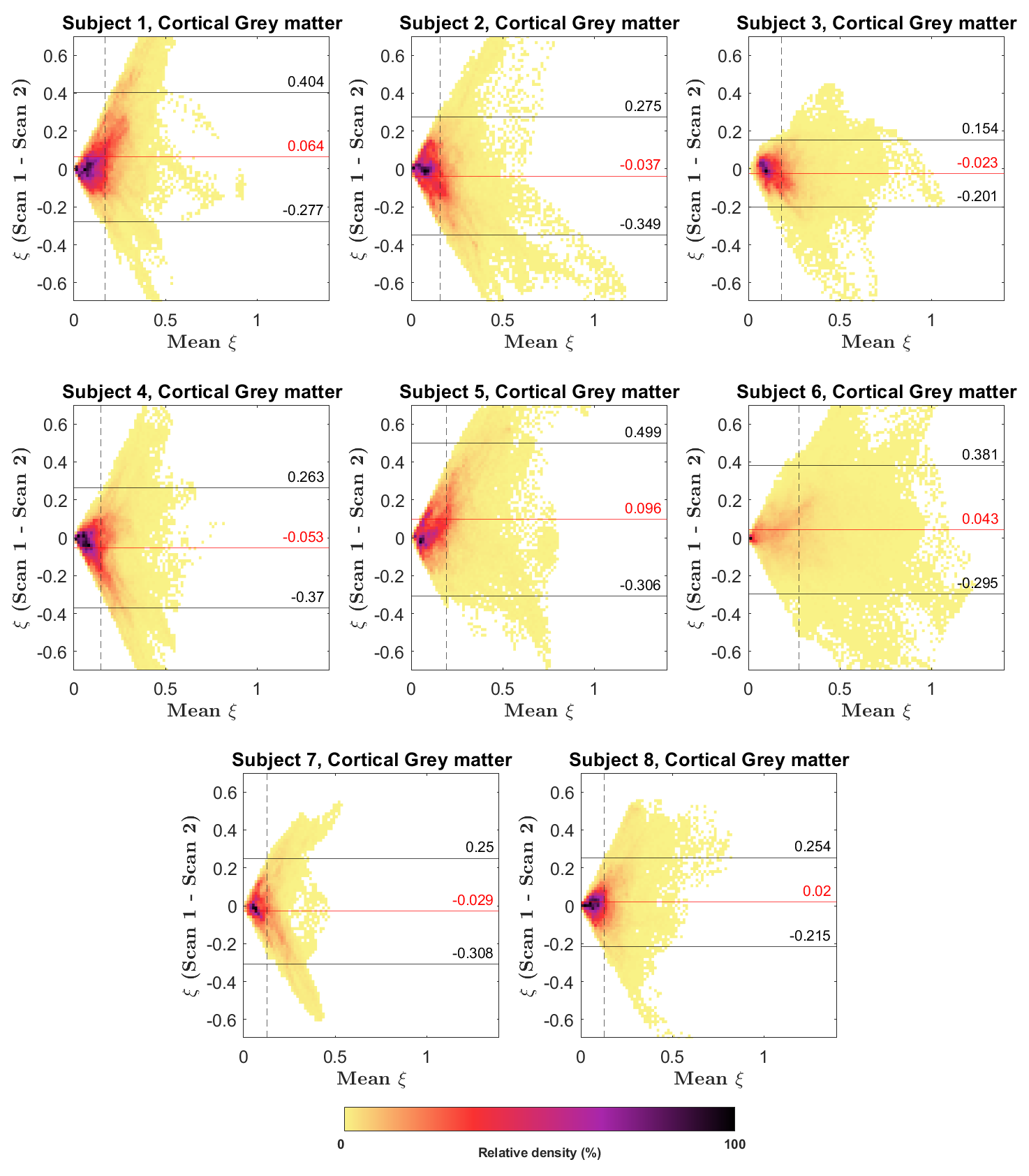


Figure S6: Bland-Altman plots for the cortical GM damping ratio in all subjects. The horizontal red line represents the bias $b$ which is the mean of the voxel-wise differences between the two measurements. The upper and lower horizontal black lines represent the upper and lower limits of agreement respectively, defined as $b\pm1.96\cdot std$. The vertical dashed line indicates the mean over all voxels for the two measurements.


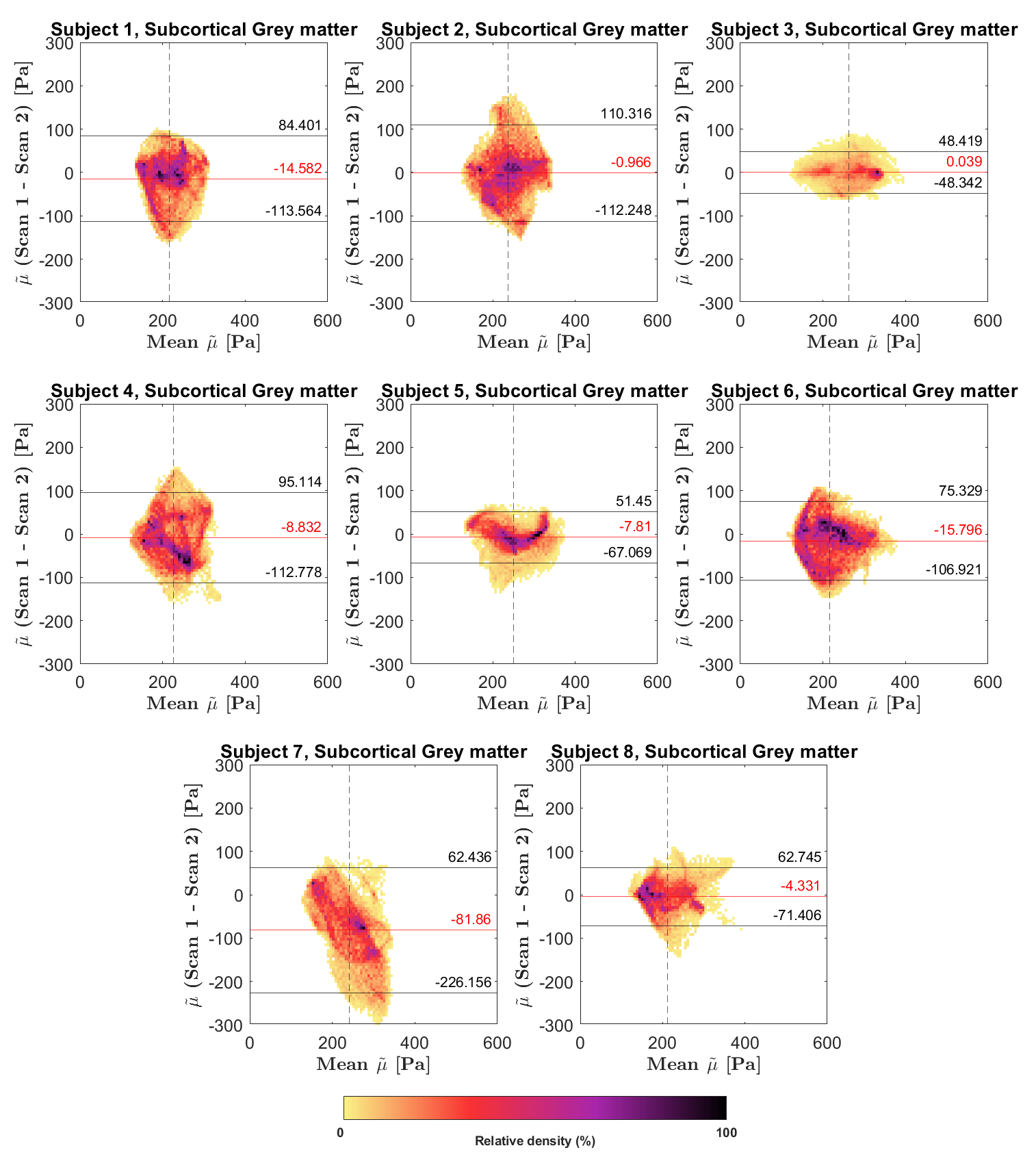


Figure S7: Bland-Altman plots for the subcortical GM shear stiffness in all subjects. The horizontal red line represents the bias $b$ which is the mean of the voxel-wise differences between the two measurements. The upper and lower horizontal black lines represent the upper and lower limits of agreement respectively, defined as $b\pm1.96\cdot std$. The vertical dashed line indicates the mean over all voxels for the two measurements.


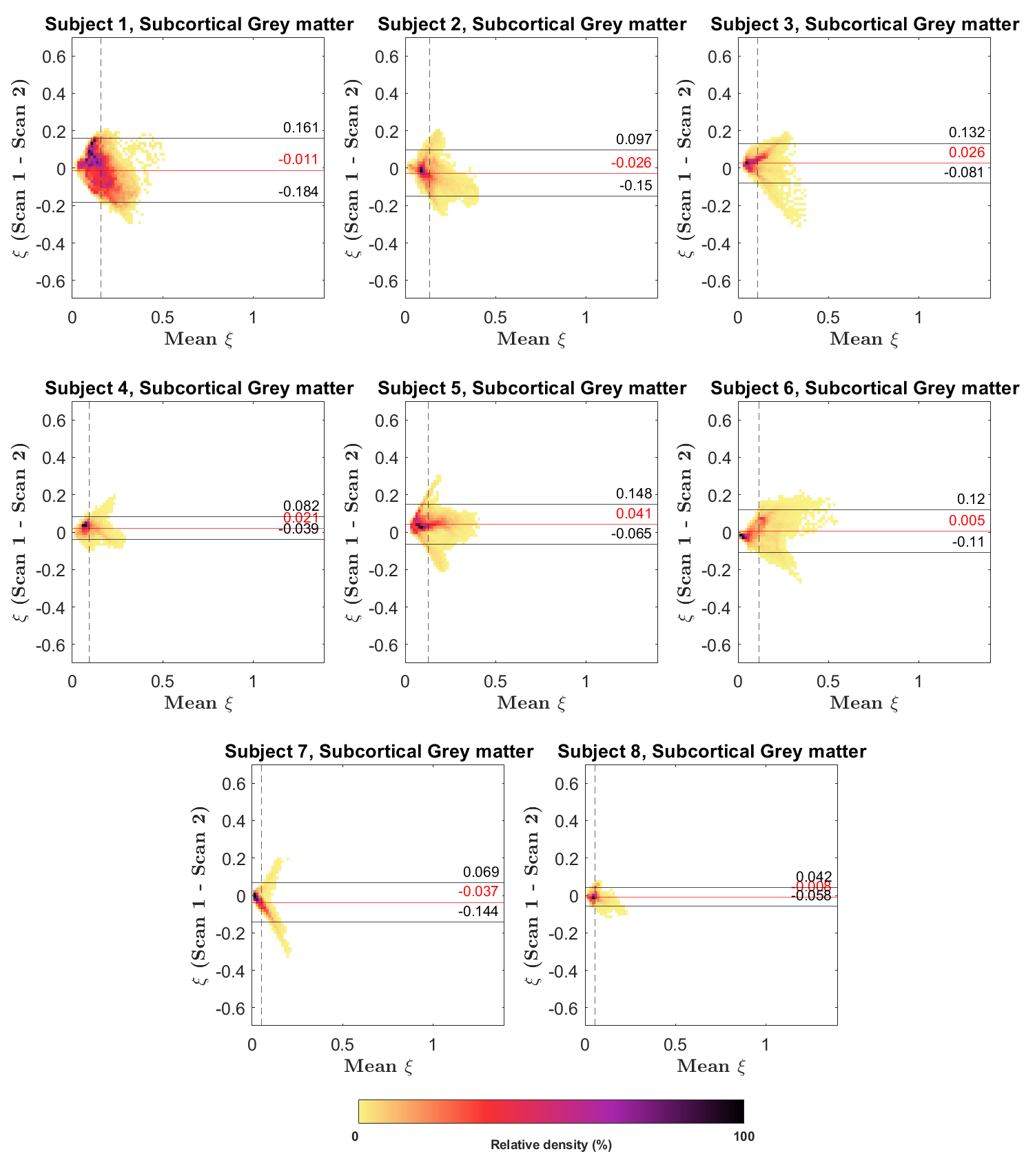


Figure S8: Bland-Altman plots for the subcortical GM damping ratio in all subjects. The horizontal red line represents the bias $b$ which is the mean of the voxel-wise differences between the two measurements. The upper and lower horizontal black lines represent the upper and lower limits of agreement respectively, defined as $b\pm1.96\cdot std$. The vertical dashed line indicates the mean over all voxels for the two measurements.

Table S1: Number of voxels in each WMT ROIs in 2 mm isotropic resolution.

|  |  |  |  |  |  |  |  |  |  |  |  |  |  |
| --- | --- | --- | --- | --- | --- | --- | --- | --- | --- | --- | --- | --- | --- |
|  |  | **SFC** | **STC** | **RMF** | **LaO** | **PRE** | **PCN** | **ITC** | **SPC** | **FSG** | **POST** | **LiO** | **CN** |
| Subject 1 | Scan 1 | 3262 | 2953 | 2056 | 1672 | 1352 | 738 | 695 | 674 | 542 | 434 | 72 | 59 |
|  | Scan 2 | 3262 | 2953 | 2056 | 1672 | 1352 | 738 | 696 | 674 | 542 | 434 | 77 | 59 |
| Subject 2 | Scan 1 | 3262 | 2953 | 2057 | 1672 | 1352 | 738 | 700 | 674 | 542 | 434 | 89 | 59 |
|  | Scan 2 | 3262 | 2953 | 2057 | 1672 | 1352 | 738 | 700 | 674 | 542 | 434 | 91 | 59 |
| Subject 3 | Scan 1 | 3262 | 2953 | 2046 | 1655 | 1352 | 738 | 696 | 674 | 542 | 434 | 76 | 59 |
|  | Scan 2 | 3262 | 2953 | 2048 | 1666 | 1352 | 738 | 690 | 674 | 542 | 434 | 65 | 59 |
| Subject 4 | Scan 1 | 3262 | 2953 | 2054 | 1672 | 1352 | 738 | 698 | 674 | 542 | 434 | 89 | 59 |
|  | Scan 2 | 3262 | 2953 | 2057 | 1671 | 1352 | 738 | 696 | 674 | 542 | 434 | 88 | 59 |
| Subject 5 | Scan 1 | 3262 | 2953 | 2056 | 1672 | 1352 | 738 | 690 | 674 | 542 | 434 | 77 | 59 |
|  | Scan 2 | 3262 | 2953 | 2056 | 1672 | 1352 | 738 | 691 | 674 | 542 | 434 | 81 | 59 |
| Subject 6 | Scan 1 | 3262 | 2953 | 2056 | 1672 | 1352 | 738 | 698 | 674 | 542 | 434 | 77 | 59 |
|  | Scan 2 | 3262 | 2953 | 2056 | 1672 | 1352 | 738 | 700 | 674 | 542 | 434 | 86 | 59 |
| Subject 7 | Scan 1 | 3262 | 2953 | 2053 | 1672 | 1352 | 738 | 686 | 674 | 542 | 434 | 75 | 59 |
|  | Scan 2 | 3262 | 2953 | 2051 | 1672 | 1352 | 738 | 670 | 674 | 542 | 434 | 69 | 59 |
| Subject 8 | Scan 1 | 3262 | 2953 | 2057 | 1672 | 1352 | 738 | 696 | 674 | 542 | 434 | 88 | 59 |
|  | Scan 2 | 3262 | 2953 | 2054 | 1672 | 1352 | 738 | 696 | 674 | 542 | 434 | 80 | 59 |

Table S2: Number of voxels in each cortical GM ROIs in 2 mm isotropic resolution.

|  |  |  |  |  |  |  |  |  |  |  |  |  |  |
| --- | --- | --- | --- | --- | --- | --- | --- | --- | --- | --- | --- | --- | --- |
|  |  | **SFC** | **STC** | **RMF** | **LaO** | **PRE** | **PCN** | **ITC** | **SPC** | **FSG** | **POST** | **LiO** | **CN** |
| Subject 1 | **Scan 1** | 8724 | 3718 | 3387 | 2966 | 2869 | 2944 | 1970 | 2685 | 2103 | 2002 | 1980 | 1033 |
|  | **Scan 2** | 8653 | 3517 | 3355 | 3121 | 2893 | 2947 | 1816 | 2702 | 2094 | 1958 | 1980 | 1015 |
| Subject 2 | **Scan 1** | 8911 | 4043 | 3634 | 3588 | 2940 | 2947 | 1972 | 2739 | 2096 | 2071 | 1980 | 1073 |
|  | **Scan 2** | 8925 | 4003 | 3633 | 3486 | 2882 | 2947 | 2024 | 2738 | 2129 | 2048 | 1979 | 1070 |
| Subject 3 | **Scan 1** | 8195 | 3327 | 3079 | 3271 | 2754 | 2947 | 1709 | 2639 | 2104 | 1900 | 1971 | 1066 |
|  | **Scan 2** | 8446 | 3300 | 3175 | 3291 | 2788 | 2947 | 2017 | 2651 | 2127 | 1932 | 1980 | 1069 |
| Subject 4 | **Scan 1** | 8597 | 3603 | 3131 | 2777 | 2822 | 2946 | 1446 | 2468 | 1854 | 1845 | 1970 | 1022 |
|  | **Scan 2** | 8416 | 3644 | 2928 | 3011 | 2691 | 2944 | 1534 | 2455 | 1875 | 1804 | 1975 | 1030 |
| Subject 5 | **Scan 1** | 8731 | 3553 | 3003 | 3014 | 2817 | 2942 | 1774 | 2433 | 1927 | 1950 | 1969 | 1019 |
|  | **Scan 2** | 8667 | 3536 | 2966 | 2833 | 2810 | 2937 | 1861 | 2383 | 2001 | 1952 | 1975 | 1006 |
| Subject 6 | **Scan 1** | 8774 | 3643 | 3371 | 3094 | 2965 | 2940 | 1726 | 2736 | 2091 | 2104 | 1960 | 1023 |
|  | **Scan 2** | 8786 | 3623 | 3340 | 3136 | 2965 | 2946 | 1873 | 2734 | 2086 | 2053 | 1971 | 1043 |
| Subject 7 | **Scan 1** | 8087 | 3420 | 2445 | 1838 | 2656 | 2922 | 1039 | 2062 | 1933 | 1735 | 1936 | 1015 |
|  | **Scan 2** | 8546 | 3063 | 2775 | 2208 | 2755 | 2940 | 965 | 2573 | 1916 | 1955 | 1948 | 1046 |
| Subject 8 | **Scan 1** | 8844 | 3987 | 3501 | 3485 | 2945 | 2947 | 1925 | 2738 | 2087 | 2112 | 1980 | 1073 |
|  | **Scan 2** | 8715 | 3849 | 3487 | 3426 | 2945 | 2947 | 2136 | 2739 | 2011 | 2064 | 1980 | 1071 |

Table S3: Number of voxels in each subcortical GM ROIs in 2 mm isotropic resolution.

|  |  |  |  |  |  |  |  |
| --- | --- | --- | --- | --- | --- | --- | --- |
|  |  | **TH** | **PU** | **CA** | **HC** | **PA** | **AM** |
| Subject 1 | **Scan 1** | 2017 | 1187 | 977 | 763 | 273 | 243 |
|  | **Scan 2** | 2017 | 1187 | 977 | 763 | 273 | 240 |
| Subject 2 | **Scan 1** | 2017 | 1187 | 977 | 751 | 273 | 205 |
|  | **Scan 2** | 2017 | 1187 | 977 | 759 | 273 | 220 |
| Subject 3 | **Scan 1** | 2017 | 1187 | 977 | 761 | 273 | 230 |
|  | **Scan 2** | 2017 | 1187 | 977 | 762 | 273 | 238 |
| Subject 4 | **Scan 1** | 2017 | 1187 | 977 | 763 | 273 | 240 |
|  | **Scan 2** | 2017 | 1187 | 977 | 763 | 273 | 241 |
| Subject 5 | **Scan 1** | 2017 | 1187 | 977 | 763 | 273 | 242 |
|  | **Scan 2** | 2017 | 1187 | 977 | 763 | 273 | 241 |
| Subject 6 | **Scan 1** | 2017 | 1187 | 977 | 763 | 273 | 236 |
|  | **Scan 2** | 2017 | 1187 | 977 | 763 | 273 | 227 |
| Subject 7 | **Scan 1** | 2017 | 1187 | 977 | 757 | 273 | 218 |
|  | **Scan 2** | 2017 | 1187 | 977 | 761 | 273 | 222 |
| Subject 8 | **Scan 1** | 2017 | 1187 | 977 | 762 | 273 | 229 |
|  | **Scan 2** | 2017 | 1187 | 977 | 763 | 273 | 234 |
